# Influenza vaccination in early Alzheimer’s disease rescues amyloidosis and ameliorates cognitive deficits in APP/PS1 mice by inhibiting regulatory T cells

**DOI:** 10.1186/s12974-020-01741-4

**Published:** 2020-02-19

**Authors:** Yunjie Yang, Zitian He, Zhiwei Xing, Zejie Zuo, Lifang Yuan, Yingying Wu, Mei Jiang, Fangfang Qi, Zhibin Yao

**Affiliations:** 1 Department of Anatomy and Neurobiology, Zhongshan School of Medicine, Sun Yat-Sen University, #74, Zhongshan No. 2 Road, Guangzhou, 510080 China; 2 Guangdong Province Key Laboratory of Brain Function and Disease, Zhongshan School of Medicine, Sun Yat-Sen University, #74, Zhongshan No. 2 Road, Guangzhou, 510080 China; 3 Teaching and Research Bureau of Surgery, Sun Yat-Sen Memorial Hospital, Sun Yat-Sen University, Guangzhou, 510120 Guangdong China; 4 Department of Anatomy and Neurobiology, Zhongshan School of Medicine, Sun Yat-Sen University, #74, Zhongshan No. 2 Road, Guangzhou, 510080 China

**Keywords:** Influenza vaccine, Alzheimer’s disease, β-Amyloid, Regulatory T cells, Microglia

## Abstract

**Background:**

Alzheimer’s disease (AD) is a neurodegenerative disorder strongly correlated with a dysfunctional immune system. Our previous results demonstrated that inactivated influenza vaccine (IIV) facilitates hippocampal neurogenesis and blocks lipopolysaccharide (LPS)-induced cognitive impairment. However, whether IIV improves cognitive deficits in an AD mouse model remains unclear. In addition, early interventions in AD have been encouraged in recent years. Here, we investigated whether IIV immunization at the preclinical stage of AD alters the brain pathology and cognitive deficits in an APP/ PS1 mouse model.

**Methods:**

We assessed spatial learning and memory using Morris water maze (MWM). The brain β-amyloid (Aβ) plaque burden and activated microglia were investigated by immunohistochemistry. Furthermore, flow cytometry was utilized to analyze the proportions of Treg cells in the spleen. A cytokine antibody array was performed to measure the alteration of cytokines in the brain and peripheral immune system.

**Results:**

Five IIV immunizations activated microglia, reduced the Aβ burden and improved the cognitive impairment. Simultaneously, the IIV-induced immune response broke peripheral immunosuppression by reducing Foxp3^+^ regulatory T cell (Treg) activities, whereas the restoration of Treg level in the periphery using all-trans retinoic acid (ATRA) blunted the protective effects of IIV on Aβ burden and cognitive functions. Interestingly, IIV immunization might increase proinflammatory and anti-inflammatory cytokine expression in the brain of APP/PS1 mice, enhanced microglial activation, and enhanced the clustering and phagocytosis of Aβ, thereby creating new homeostasis in the disordered immune microenvironment.

**Conclusions:**

Altogether, our results suggest that early multiple IIV immunizations exert a beneficial immunomodulatory effect in APP/PS1 mice by breaking Treg-mediated systemic immune tolerance, maintaining the activation of microglia and removing of Aβ plaques, eventually improving cognitive deficits.

## Introduction

Alzheimer’s disease (AD) is among the most prevalent forms of dementia and is characterized by the extracellular accumulation of senile plaques consisting of amyloid-β (Aβ), intracellular aggregation of neurofibrillary tangles (NFTs) composed of hyperphosphorylated tau, chronic neuroinflammation, gliosis, and progressive cognitive decline; however, effective treatments are lacking [1–3].

Neuroinflammation is a complex and uncoordinated inflammatory process involving innate immune cells in the central nervous system and peripheral circulation [4–6]. Mounting evidence gathered over previous decades suggests that the AD-immune system is chronically activated with AD pathology [7–9]. Accordingly, studies investigating the effects of anti-inflammatory and immunosuppressive treatments in AD have indeed exhibited efficacy in the preclinical stage; however, all clinical trials have failed [10–12]. Other studies have suggested that systemic immunosuppression, which has shown some effectiveness, might impair the normal immunity required to fight cerebral pathology due to the lack of immune surveillance function in AD [13–16]. Our previous study showed that Bacillus Calmette-Guerin (BCG)-induced effective immunomodulatory effects, promoted neurogenesis under physiological conditions, mitigated systemic immune suppression, and improved cognitive deficits in AD, without Aβ plaque clearance [17]. However, whether multiple early vaccinations with inactivated influenza vaccine (IIV) could relieve the brain pathology and memory deficits in AD remains to be determined.

Influenza virus pandemics cause millions of mortality annually, especially among elderly individuals aged over 65 years whose immune system is detrimentally affected by aging [18]. Adults aged 65 years or older, whose immune function is poor due to aging, have an increased susceptibility to influenza [19–21]. Thus, IIV vaccination has been advocated in older people to prevent the flu in China [22]. We reported that IIV, which is a nonspecific system immune mediator, facilitates neurogenesis and behavioral functions in pregnant mice and offspring, recruits peripheral T cells to the choroid plexus (CP), and shifts microglia to an M2-like phenotype [23, 24].

Regulatory T cells (Tregs) act as system immunomodulators, which play an important role in maintaining immune homeostasis by suppressing excessive immune responses [25, 26]. The number of interleukin (IL)-10-producing CD4^+^ T cells in AD patients is higher than that in healthy controls [27]. Furthermore, breaking immune tolerance by suppressing Tregs ameliorates cerebral Aβ burden and AD pathology [13]. Evidence suggests that the ability of microglial activation surrounding Aβ plaques to protect against the occurrence of AD may decrease with age and disease progression because of impaired microglial function [28, 29]. However, the restoration of microglial responses to Aβ through the modulation of peripheral immunity may be beneficial [13].

Therefore, to further reveal the relationship between Tregs in the periphery and the microglial response in AD mice, we explored the hypothesis that the inhibition of Treg activities by IIV immunization during the early stage of AD may improve cognition performance in amyloid precursor protein (APP)/presenilin 1 (PS1) mice. Our findings demonstrate that early preventive IIV immunization activates the immune system, enhances the microglial response to plaques, rebalances the disordered cerebral immune milieu and ultimately alleviates cognitive deficits in APP/PS1 mice. Additionally, we conclude that targeting Tregs to restore the normal phagocytosis of microglia is a promising therapeutic strategy for AD.

## Materials and methods

### Animals

Two-week-old male APP/PS1 (APPswePSEN1dE9) transgenic mice, on a C57BL/6 background, and age-matched wide-type mice obtained from the same source, were purchased from the Laboratory Animal Center of Sun Yat-Sen University (Guangzhou, China) or the Nanjing Biomedical Research Institute of Nanjing University (Nanjing, China). The promoters of both transgenes in the APP/PS1 transgenic mice co-expressing the human KM670/671L-mutated APP gene and the M146L-mutated PS1 gene are controlled by the mouse prion protein readthrough transcript (Prn). All animals were housed under temperature- and humidity-controlled conditions and maintained in a 12-h light/dark cycle environment for 10 weeks before further treatment. All experimental protocols complied with the regulations of the Institutional Animal Care and Use Committee of Sun Yat-Sen University.

### Influenza vaccination

**Fig. 1:**
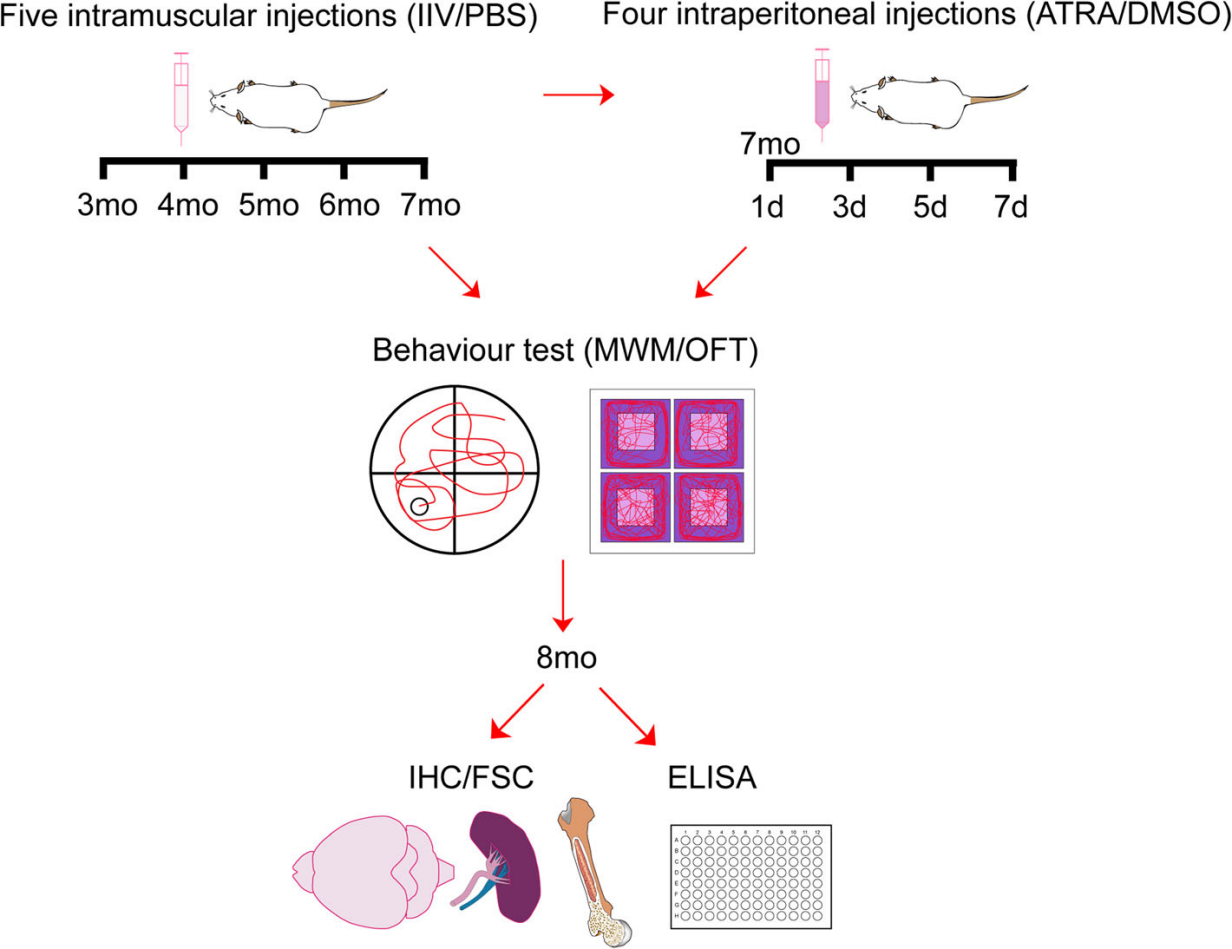
Experimental protocol. Three-month-old APP/PS1 mice or C57BL/6 mice received five injections. The animals were intramuscularly (i.m.) injected with IIV or PBS every 4 weeks over a 5-month period. Three weeks after the final injection, the MWM and OFT were conducted to assess the spatial learning and memory and spontaneous locomotor activity of the mice. Then, tissues were collected for the subsequent experiments at the age of 8 months. At the age of 7 months, the AD+IIV mice were i.p. injected with ATRA or DMSO 5 times every other day and tested as previously described

Three-month-old APP/PS1 mice or C57BL/6 mice were intramuscularly injected with split IIV or sterilized phosphate buffer saline (PBS) in the quadriceps femoris at a single dose of 3 μg/mouse every 4 weeks for a total of five times (Fig. 1). The effective constituents of IIV included A/California/7/2009 (H1N1) pdm09-like virus, A/Hong Kong/4801/2014 (H3N2)-like virus and B/Brisbane/60/2008-like virus, whereas the excipients included NaH2PO4, Na2HPO4, and NaCl. The vaccines were purchased from the Center for Disease Prevention and Control of Guangdong (CDC, China) and produced by Changchun Research Institute of Biological Products.

### ATRA administration

Seven-month-old IIV-treated APP/PS1 mice (AD+IIV mice) were intraperitoneally (i.p.) injected with ATRA (Sigma) dissolved in dimethyl sulfoxide (DMSO) at 8 mg/kg every 2 days within 1 week [13] for a total of four injections. DMSO was injected in the vehicle group using a similar approach (Fig. 1). Similarly, 7-month-old APP/PS1 mice and WT mice were intraperitoneally (i.p.) injected with ATRA (Sigma) dissolved in dimethyl sulfoxide (DMSO) at 8 mg/kg every 2 days within 1 week [13] for a total of four injections. DMSO was injected in the vehicle group using a similar approach.

### Open field test (OFT)

The animals were individually placed in the center of an open field chamber (50 × 50 × 50 cm) and allowed to explore for 10 min. The spontaneous locomotor activity and movements in the arena were monitored by an overhead camera and tracked with a TopScan TM 2.0 system (Clever Sys. Inc.). The distance traveled in the open field and outer and center arenas, the duration spent in the center arena, and the number of entrances into the center arena were automatically recorded.

### Morris water maze (MWM)

The MWM was used to assess the spatial learning and memory performance of all experimental animals. The experiments were performed in a 100-cm-diameter tank filled with opacified water maintained at 21.5 ± 1.5 °C. The maze was virtually divided into four quadrants, and a 10-cm-diameter platform was placed in the center of the third quadrant 1 cm below the water surface. During the task acquisition phase, the mice were placed in the maze facing the wall and allowed to swim for 60 s, this phase consisted of four trials per day over 5 consecutive days. The escape latency and swimming distance to find the platform were recorded. If the mice were unable to reach the platform within 60 s, they were manually placed onto the platform, where they remained for 15 s. During the spatial probe trials, which were performed on day 6, the platform was removed from the maze, and the mice were allowed to swim for 60 s. The data were recorded by a computerized tracking system (MT-200, Chengdu, China).

### Tissue preparation

After the behavioral tests, all mice were deeply anesthetized. Serum samples were collected by removing the eyeball and stored at − 80 °C. Then, the mice were transcardially perfused with 50 ml cold normal saline. The brains were immediately removed, and dissected into two hemispheres from the median sagittal suture on ice. One hemisphere was fixed in 4% paraformaldehyde for the histological analysis, while the hippocampus and cortex were quickly isolated from the other hemisphere, snap-frozen in liquid nitrogen, and stored at − 80 °C for the subsequent biochemical analysis.

### Brain protein extraction

The snap-frozen mouse hemi-brains were homogenized and extracted at 4 °C in RIPA buffer (50 mM Tris-HCl, pH 7.4, 150 mM NaCl, 1% Triton X-100, 1% sodium deoxycholate, 0.1% SDS), 1% phosphatase inhibitors (Sigma-Aldrich), and 1% protease inhibitors (Sigma-Aldrich) using TissueRuptor (QIAGEN) and centrifuged for 30 min at 100,000×*g* (Beckman, Optima L-100XP). The supernatant was collected as the RIPA-soluble fraction, and the pellet was extracted in 2% SDS, 50 mM Tris-HCl, pH 7.4. The supernatants were collected as the SDS-soluble fraction. Then, the homogenate pellet was extracted in cold formic-acid (FA) and centrifuged at 100,000×*g* for 1 h at 4 °C. The supernatant was neutralized with 200 mM Tris-HCl, pH 7.5, collected as the FA-extracted insoluble fraction and stored at − 80 °C.

### Immunohistochemistry and quantitative analyses

The post-fixed brain hemispheres were frozen in 2-methylbutane and sectioned coronally at 40 μm using a freezing microtome (Leica SM2000R) after 2 days of cryoprotection in 30% sucrose/phosphate buffer (PB). The sections were stained with primary antibodies in blocking buffer by overnight incubation at 4 °C after washing 3 times with PBS and blocking in 1% BSA at 37 °C for 30 min. After rinsing 3 times with PBS, the slices were incubated with immunofluorescent secondary antibodies at a dilution of 1:400 for 2 h at 37 °C and then washed again. Hoechst (1:1000; H33258, Invitrogen) was applied for 1 min to counterstain the cell nuclei. The primary antibodies used included mouse anti-Aβ1-42 (1:1000, A5213, Sigma-Aldrich), rabbit anti-ionized calcium-binding adapter molecule 1 (Iba-1; 1:1000, 019-19741, Wako), and rat anti-CD68 (1:400, MCA1957, Bio-Rad). The secondary antibodies used included Alexa Fluor 647 donkey anti-mouse (1:400, Invitrogen), Alexa Fluor 488 donkey anti-rat (1:400, Invitrogen), and Alexa Fluor 555 goat anti-rabbit (1:400, Invitrogen).

For the image analysis, an LSM 780 confocal laser scanning microscope (Zeiss) was used to capture the images of each section using the same parameters to avoid potential technical artifacts. The measurements were performed at a continuous equidistance of five coronal slices spaced 240 μm apart. For the quantification of the staining areas in the area of interest in each image, ImageJ software (NIH) was used.

### Three-dimensional reconstruction of confocal images

High-magnification confocal z-stack images (captured at × 63 zoom in 1.6 magnification under a Zeiss LSM780) of amyloid plaques phagocytosed by activated microglia were converted to three-dimensional images using the surface and colocalization functions in Imaris software (Bitplane, version 8.4) to colocalize, reconstruct the surface, and quantify the volume of Iba-1, CD68 and Aβ. The immunoactivity of Iba-1 cells in close proximity to Aβ plaques was quantified by calculating the volume ratio of Iba-1 within the field. The colocalization of Iba-1 and CD68 is regarded as phagolysosomes within microglia and the colocalization of Aβ and microglial phagolysosomes is regarded as Aβ within phagolysosomes. Therefore, the volume of Aβ plaques in CD68^+^ phagolysosomes was normalized to the microglia volume and total Aβ volume within the field to calculate the Aβ internalization ratio.

### Quantitative enzyme linked immunosorbent assay (ELISA) of Aβ and serum IL-10

The concentrations of cerebral soluble and insoluble Aβ_1-40_ and Aβ_1-42_ were measured using ELISA kits (BioLegend; cat. no. 842301 and cat. no. 842401, respectively) according to the manufacturer’s protocol. The total protein concentrations were quantitated using a bicinchoninic acid (BCA) protein reagent kit (P0012S, Beyotime) before the levels of Aβ were detected. Furthermore, the concentration of serum interferon (IL)-10 was measured using an IL-10 ELISA kit (Arigo, cat. no. ARG80200) according to the manufacturer’s protocol.

### Flow cytometry

The spleens were isolated from the animals immediately after perfusion with ice-cold normal saline, immersed in alcohol and mashed with the plunge of a syringe. Then, 1× ACK (ammonium chloride potassium)-lysing buffer was added to remove erythrocytes and generate a single-cell suspension. For the Treg staining, a Mouse Regulatory T Cell Staining Kit #2 (eBioscience, 88-8118) was used. The samples were stained according to the manufacturer’s protocols. For the intracellular staining of interferon (IFN)-γ, IL-4 and IL-17A, the cells were incubated with phorbol 12-myristate 13-acetate (PMA, 50 ng/ml; Sigma), brefeldin-A (BFA, 10 μg/ml; Sigma) and ionomycin (250 ng/ml; Sigma) for 5 h. The bone marrow cells were washed out from both femurs of the mouse via a syringe with cold Hank’s balanced salt solution (HBSS) and treated with 1× ACK-lysing buffer to remove erythrocytes and prepare single-cell suspensions. The following fluorochrome-labeled monoclonal antibodies were purchased from BioLegend and eBioscience: APC-conjugated anti-CD3 FITC-conjugated anti-CD8, BV421 or FITC-conjugated anti-CD4, PE-conjugated anti-CD25, APC-conjugated anti-FoxP3, PE-CY7-conjugated anti-IFN-γ, PE-conjugated anti-IL-4, FITC-conjugated anti-IL-17A, PE-conjugated anti-CD45 and FITC-conjugated anti-CD11b. To identify the populations of interest and exclude other populations, specific negative control, positive control and isotype control groups, and single-stained samples of each tissue were used in each experiment. All samples were filtered through 75-μm nylon meshes before the analysis, assessed using a flow cytometer (Beckman CytoFLEX S) and analyzed with FlowJo software.

### Cytokine antibody array

The cytokine profiles in the serum and brain lysates from the WT, APP/PS1 (AD), and AD+IIV groups (*n* = 1, which is a mixture of four serum samples or brain tissue lysates in each group) were analyzed using a RayBio Mouse Cytokine Antibody Array (cat. QAM-CAA-4000) and tested according to the experimental protocols. The relative signal intensity of the indicated cytokines by a Gene Ontology (GO) functional enrichment analysis is presented in the trend graph.

### Statistical analysis

The statistical analyses were performed using SPSS 20.0 software for Windows (IBM). The escape latency data were analyzed using two-way repeated-measures ANOVA, and LSD post hoc comparisons were used for the follow-up pairwise comparisons. One-way ANOVA was used to compare two or more groups, followed by an LSD post hoc comparison. Student’s *t* test was used for the remaining statistical analyses. As the basis of all appropriate tests, Shapiro-Wilk test and Levene’s test were performed to test normality and homogeneity of variance. All data are presented as the mean ± SEM, and *P* < 0.05 was considered indicative of a significant difference.

## Results

### IIV treatment ameliorates cognitive deficits in APP/PS1 mice

**Fig. 2:**
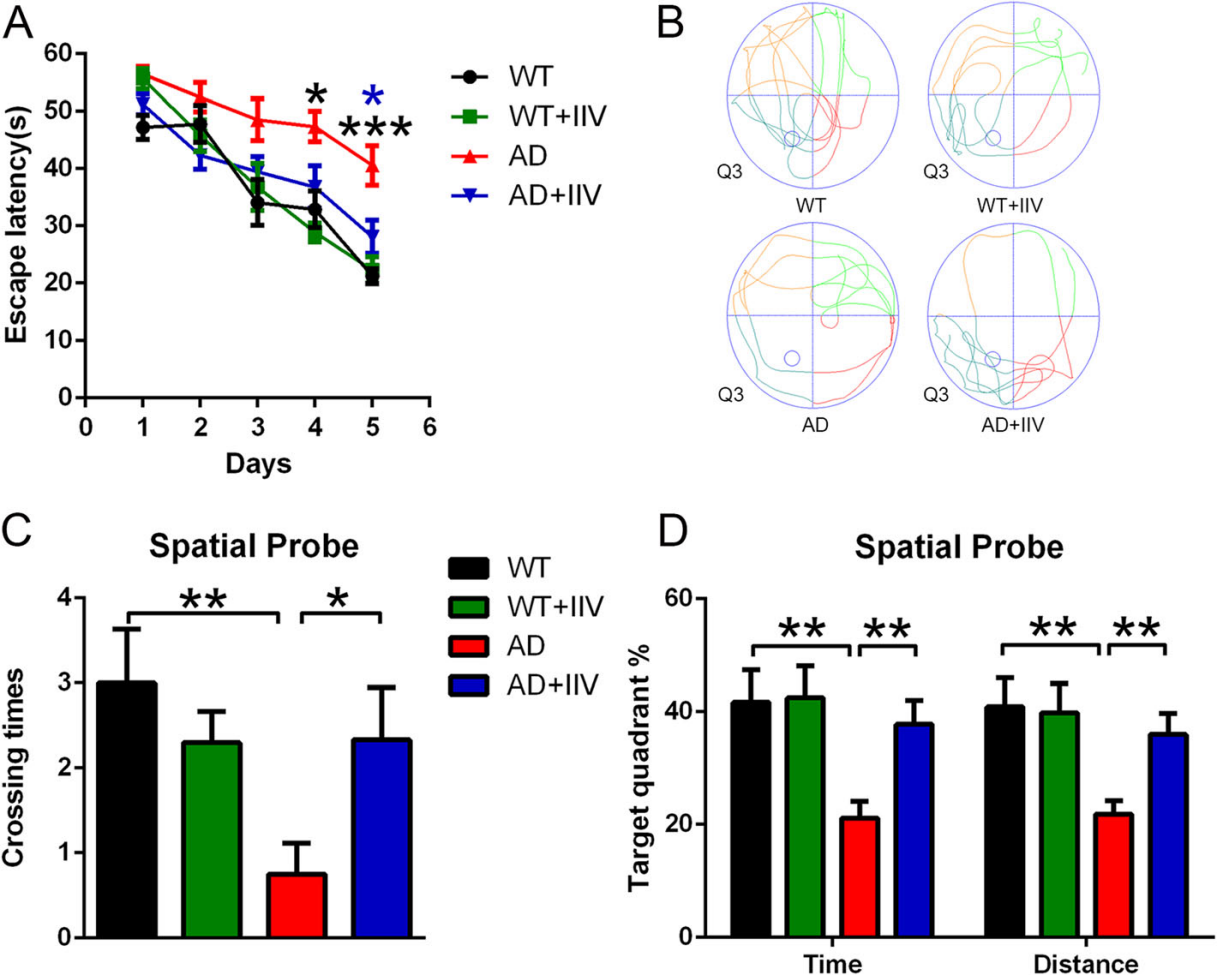
Protective effects of IIV on cognitive function in APP/PS1 animals. **a** MWM analysis of the escape latency (**a**) in the WT mice (*n* = 8), WT+IIV mice (*n* = 14), AD mice (*n* = 15) and AD+IIV mice (*n* = 16) (mean ± SEM, two-way repeated-measures ANOVA and LSD post hoc test, *P < 0.05, ***P < 0.001). **b–d** Average numbers of platform crossings (**c**), percentage of time spent and distance traveled (**d**) in the target quadrant in each group and their representative traces (**b**) during the spatial probe phase (mean ± SEM, one-way ANOVA, and LSD post hoc analysis; *P < 0.05, **P < 0.01)

To explore whether the nonspecific immunomodulator IIV affects the spatial learning and memory performance of APP/PS1 mice, 3-month-old APP/PS1 mice received 5 IIV intramuscular injections (AD+IIV mice) every 4 weeks over the course of 5 months (Fig. 1). Three weeks after the final injection, the spatial learning and memory abilities were compared between the groups. Compared with the WT mice, the APP/PS1 mice exhibited a severe cognitive impairment, but IIV significantly improved such deficits (Fig. 2). During the acquisition phase, the escape latency, while searching for the platform in the AD+IIV mice was remarkably lower than that in the APP/PS1 controls (AD mice) (Fig. 2a). During the spatial probe phase, the AD+IIV mice had a significantly greater number of passes across the platform than the AD mice (Fig. 2b, c), and the percentages of time spent and distance traveled in the target quadrant were significantly increased after IIV treatment in the AD mice (Fig. 2d). In addition, IIV had no significant effect on locomotor activity in the APP/PS1 mice and WT mice in the OFT (Fig. S1). Furthermore, to investigate the side effects of IIV, we recorded the weights and body temperatures 1 day after each injection and found no obvious differences between the AD+IIV mice and AD mice (data not shown). Taken together, our results indicate that early multiple IIV interventions effectively alleviate cognitive dysfunction in APP/PS1 mice.

### IIV treatment attenuates the cerebral Aβ plaque burden in APP/PS1 mice

**Fig. 3:**
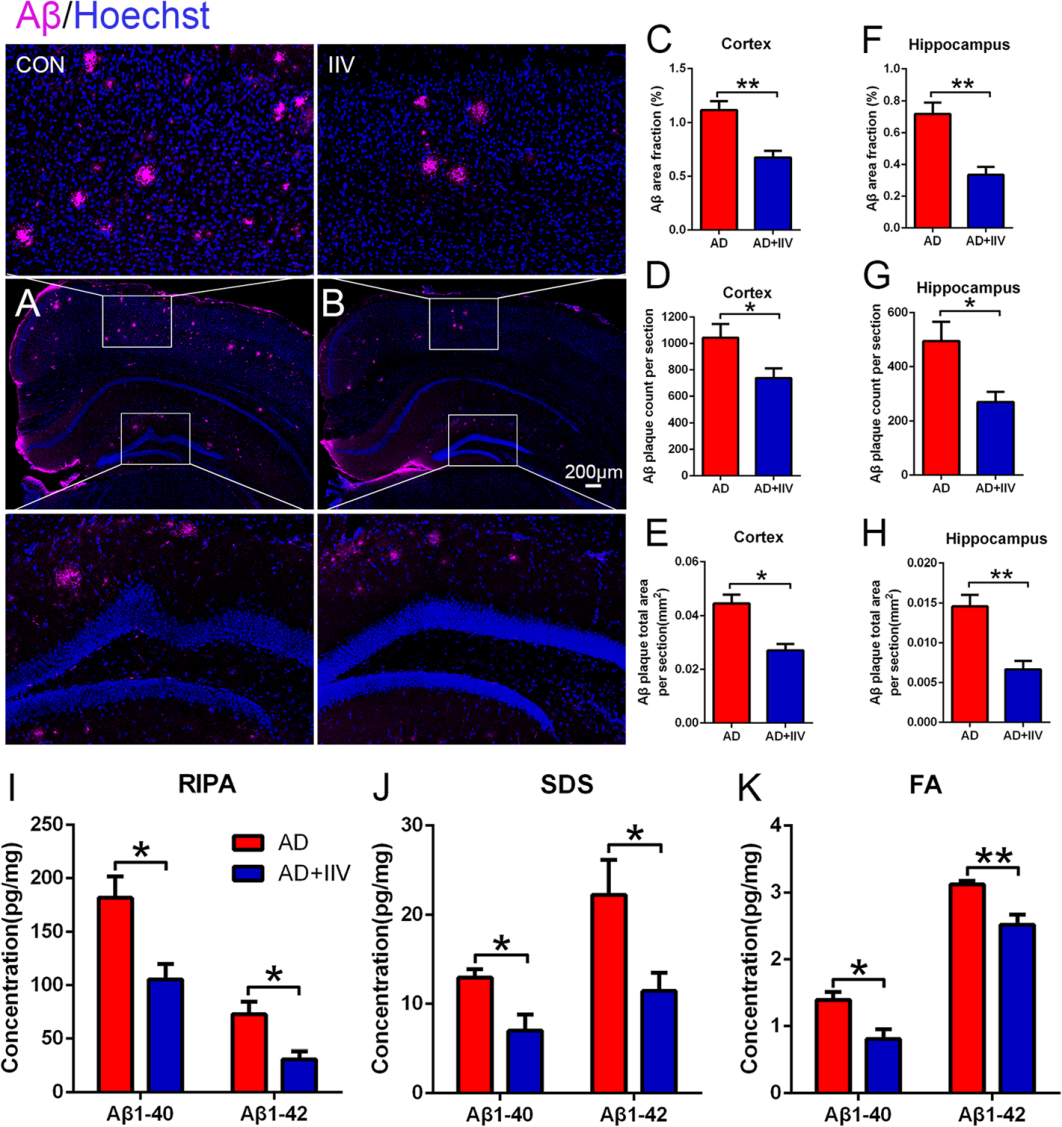
IIV treatment decreased Aβ deposition in APP/PS1 mice. **a**, **b** Representative microscopy images of the cortex and hippocampus of AD (**a**) and AD+IIV mice (**b**) staining for Aβ plaques (purple) and with Hoechst for nuclear staining (blue) (scale bar, 200 μm). **c**–**f** Quantification of the percentage of the surface area, numbers and total area of Aβ plaques was performed in five equidistant slices separated by 240 μm per animal (*n* = 9, mean ± SEM, Student’s *t* test, *P < 0.05, **P < 0.01). **i**–**k** Biochemical analysis of soluble and insoluble Aβ_1-40_ and Aβ_1-42_ extracted from the cerebral cortex with RIPA (**i**), SDS (**j**), and FA (**k**) from 8-month-old APP/PS1 mice by ELISA (*n* = 5, mean ± SEM, Student’s *t* test, *P < 0.05, **P < 0.01)

We previously reported that BCG vaccination improved cognitive deficits in AD, but without Aβ plaque clearance [17]. To determine the effect of IIV on Aβ pathology in APP/PS1 mice, we examined the area and percentage of Aβ burden in cerebral sections. Post-fixed hemispheres were used for further analysis of the cerebral Aβ pathology by immunohistochemistry. IIV significantly decreased cerebral Aβ plaque deposition in both the cortex and hippocampus (Fig. 3a–h), which are the two main areas exhibiting plaque pathology in APP/PS1 mice. Similarly, the quantification analysis of soluble and insoluble Aβ_1-40_ and Aβ_1-42_ extracted from the cerebral cortex by RIPA, SDS, and FA buffer using ELISA supported these results (Fig. 3i–k). Collectively, our data suggest that IIV reduces the cerebral Aβ plaque load, which may be an important cause of the observed cognitive improvement.

### Activity and phagocytosis of microglia are enhanced in IIV-treated APP/PS1 mice

**Fig. 4:**
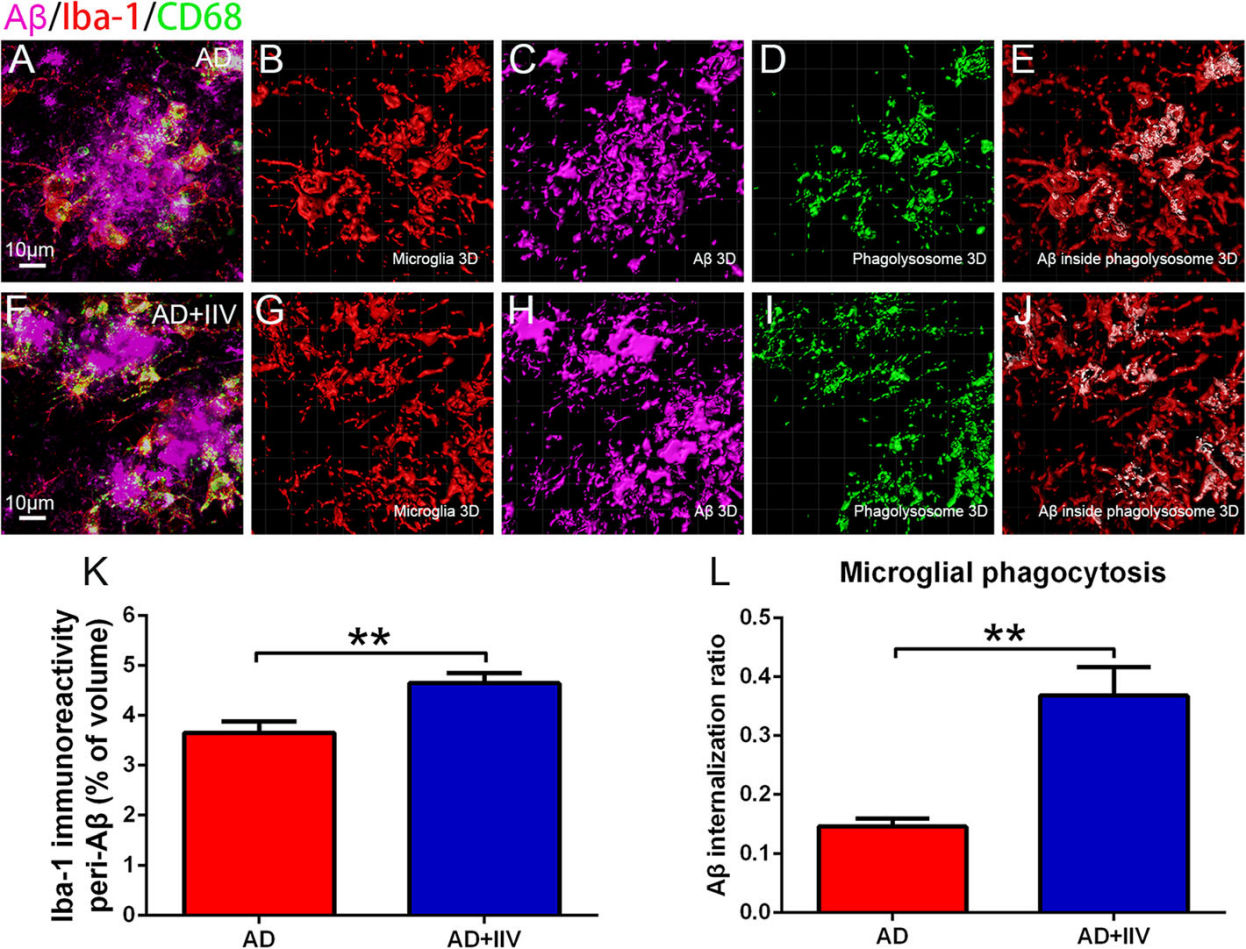
IIV treatment enhanced microglial phagocytosis ability in APP/PS1 mice. **a**, **f** Representative merged microscopy images of Aβ plaques phagocytosed by microglia in AD and AD+IIV mice stained for microglia (Iba1, red), Aβ (Aβ, purple) and phagolysosomes (CD68, green). **b**–**e**, **g**–**j** 3D reconstruction of red microglia (**b**, **g**), purple Aβ (**c**, **h**) and green phagolysosomes in microglia (colocalization of CD68 and Iba1, **d**, **i**) and merged images of transparent microglia and white amyloid plaques within microglial phagolysosomes (colocalization of Aβ plaques and microglial phagolysosomes, **e**, **j**) (scale bar, 15 μm). **k** Quantification of the percentages of Iba-1 immunostaining volume in close vicinity to Aβ plaques within the field in the AD and AD+IIV groups (*n* > 8, mean ± SEM, Student’s *t* test, **P < 0.01). **l** Assessment of the fractions of Aβ volume within microglial phagolysosomes, normalized to activated microglia, namely, the Aβ internalization ratio, revealed a threefold increase in Aβ internalization in the AD+IIV group compared with that in the AD group (*n* > 8, mean ± SEM, Student’s *t* test, **P < 0.01)

Microglia are brain-resident myeloid cells that act as immunomodulators by surveilling the cerebral immune milieu, maintaining homeostasis, and engulfing and degrading misfolded proteins [4, 30]. However, in several neurodegenerative diseases, including AD, microglia are chronically activated, and microglia-mediated clearance mechanisms are compromised [4, 31]. Therefore, we assessed whether the systemic immunity induced by IIV immunization restored microglial phagocytic ability and amyloid plaque clearance. Imaris software was utilized to investigate microglial immunoreactivity and amyloid plaques engulfed by activated microglia using 3D high-magnification images. IIV facilitated the recruitment of microglia in close vicinity to Aβ plaques (Fig. 4a–k) and simultaneously increased the proportions of Aβ plaques engulfed by microglial phagolysosomes (Fig. 4 a–j, l). Altogether, these findings suggest that multiple IIV vaccinations during the early stage of AD enhance microglial activation and recruitment to the vicinity of Aβ plaques and Aβ phagocytosis.

### IIV treatment alters peripheral immune profiles in APP/PS1 mice

**Fig. 5:**
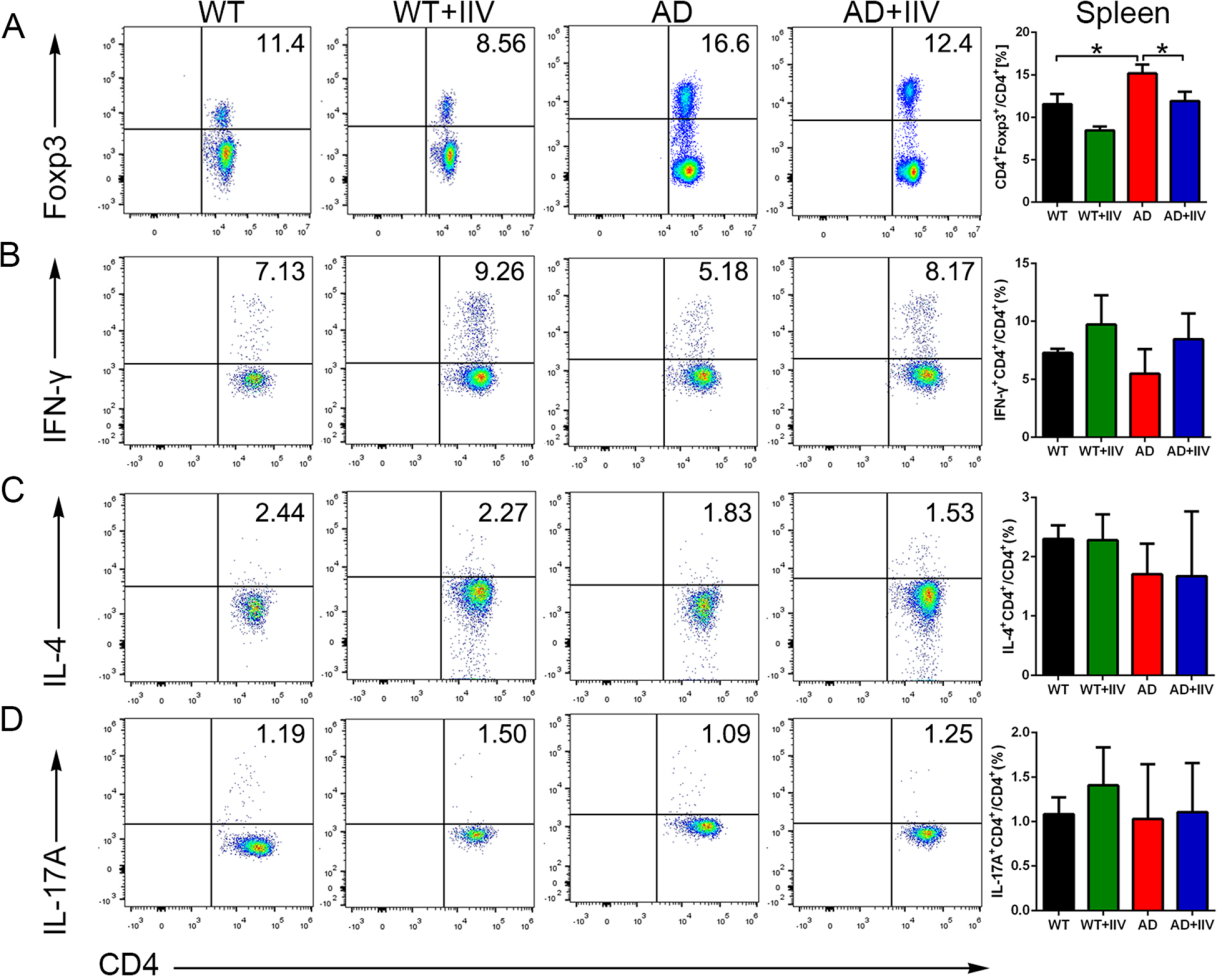
IIV treatment altered T cell homeostasis and reversed excessive CD4^+^Foxp3^+^ Tregs activity in APP/PS1 mice. **a** Representative flow cytometry plots and quantitative analysis of the proportions of CD4^+^Foxp3^+^ Treg splenocytes reflecting that the frequencies of Tregs in the AD mice were higher than those in the WT mice, while the IIV treatment reversed the excessive Treg accumulation (*n* > 10, mean ± SEM, one-way ANOVA and LSD post hoc test, *P < 0.05). **b**–**d** Intracellular staining of CD4^+^ splenocytes producing IFN-γ (**b**), IL-4 (**c**), and IL-17A (**d**) is presented as flow cytometry plots, and the quantitative analysis of each cytokine is shown on the right (*n* > 5, mean ± SEM, one-way ANOVA and LSD post hoc test)

The peripheral immune response is critically involved in preserving brain homeostatic functions and AD pathogenesis [32]. Therefore, to better understand the specific effects of IIV on the peripheral immune system, we analyzed single-cell suspensions of spleen tissues from AD+IIV mice, AD mice, WT+IIV mice and WT mice utilizing flow cytometry. The proportions of CD4^+^CD25^+^Foxp3^+^ Tregs in the spleen in AD mice were obviously higher than those in the age-matched WT mice, but normalized to the levels in the WT mice after IIV treatment (Fig. 5a). Although the ratio of CD4^+^IFN-γ^+^ T (T helper 1, Th1) cells showed an increasing trend in AD+IIV mice, the ratio did not significantly differ from that in AD mice (Fig. 5b). Additionally, no obvious changes were observed in the ratios of CD4^+^IL-4^+^ T (T helper 2, Th2) and CD4^+^IL-17A^+^ T (T helper 17, Th17) cells (Fig. 5c, d). Moreover, IIV treatment did not have a significant impact of on the CD8+ T cell compartment (data not shown). Notably, IIV stimulation reversed the decrease in the frequency of CD45^+^CD11b^+^ monocyte-derived macrophages (mo-MΦ) in the bone marrow (BM) in the APP/PS1 mice relative to that in the WT mice (Fig. S2A, B), revealing that the mo-MΦ counts in the BM increase as the Foxp3^+^ Tregs quantities decrease after IIV treatment. Therefore, our present study suggests that IIV-related peripheral immune profiles, at least those of Tregs and mo-MΦ, modify AD pathogenesis and cognitive function.

### Enhancing immunosuppression by ATRA prevents IIV-induced protection against Aβ pathology and cognitive deficits

**Fig. 6:**
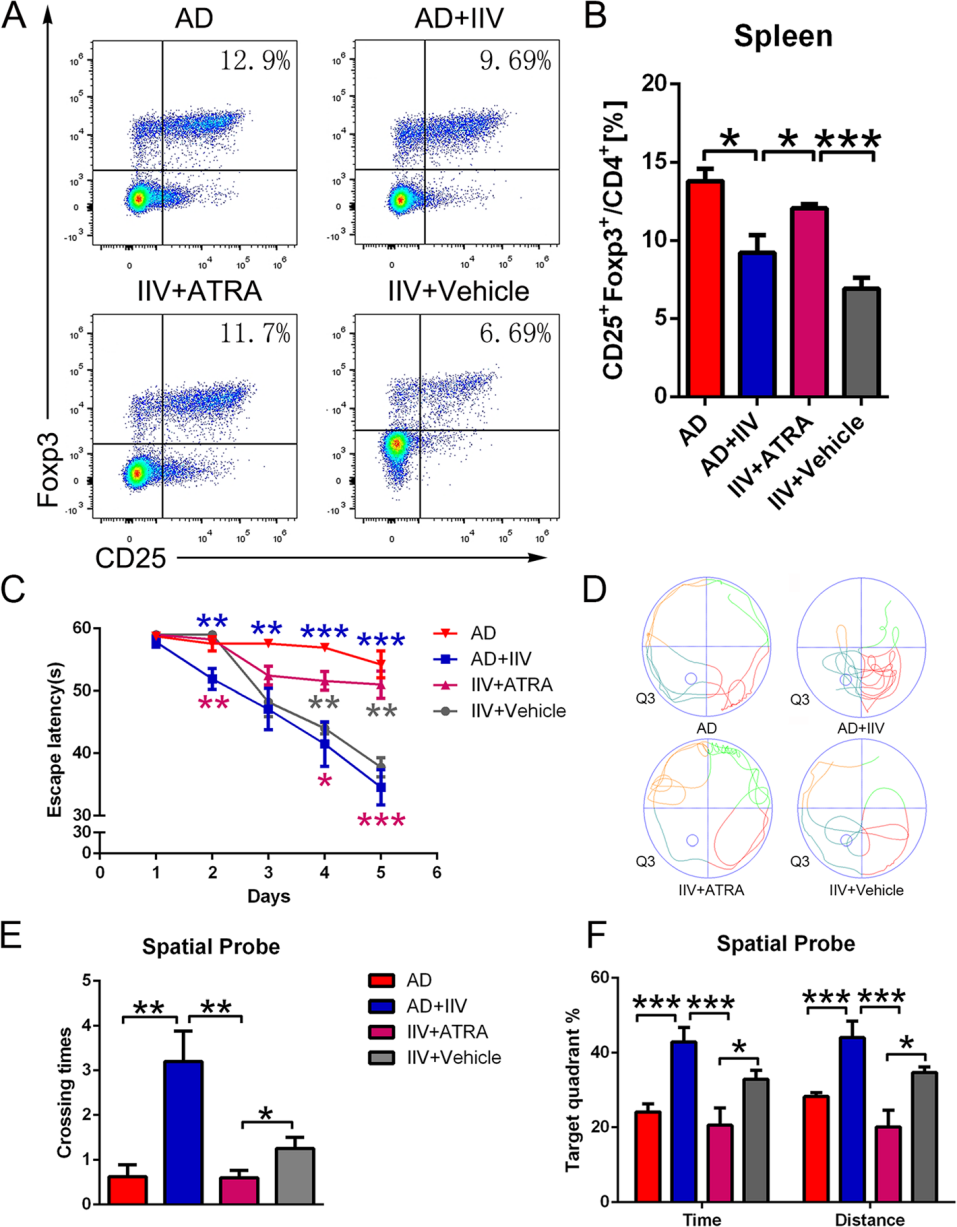
Enhanced immunosuppression impaired IIV-induced cognitive improvements in APP/PS1 mice. **a**, **b** Flow cytometric analysis of the proportions of CD4^+^CD25^+^Foxp3^+^ Treg splenocytes in the IIV+ATRA or IIV+Vehicle (DMSO) mice. Representative flow panels and quantitative analysis indicated that ATRA increased the percentages of CD4^+^CD25^+^Foxp3^+^ Tregs, compared with AD+IIV and IIV+Vehicle mice (*n* > 5, mean ± SEM, one-way ANOVA and LSD post hoc test, *P < 0.05, ***P < 0.001). **c**–**f** Escape latency in the MWM (**c**) in the AD (*n* = 8), AD+IIV (*n* = 8), IIV+ATRA (*n* = 6) and IIV+Vehicle (*n* = 6) mice (mean ± SEM, two-way repeated-measures ANOVA and LSD post hoc test, *P < 0.05, **P < 0.01, ***P < 0.001). The purple asterisk represents the comparison of the AD+IIV and IIV+ATRA mice. The blue asterisk indicates the comparison of the AD mice and AD+IIV mice. The gray asterisk represents the comparison of the IIV+ATRA and IIV+Vehicle mice. Average numbers of platform crossings (**e**), ratio of time spent and distance traveled (**f**) and representative trajectories (**d**) in the target quadrant in each group during the spatial probe phase (one-way ANOVA and LSD post hoc analysis; *P < 0.05, **P < 0.01, ***P < 0.001)

Recently, several studies investigating Treg function in AD pathology yielded different results [13, 33, 34]. Therefore, whether Tregs exert neuroprotective or immune suppressive effects in AD mouse models remains to be fully elucidated. To determine the role of Tregs in IIV-induced cognitive rehabilitation, we used a pharmacological method to induce a transient systemic Treg expansion in splenocytes in APP/PS1 mice, via an intraperitoneal injection of ATRA [35]. We found that the frequency of CD4^+^CD25^+^Foxp3^+^ Tregs in the spleens of AD+IIV mice after ATRA treatment (IIV+ATRA) was significantly higher than that in the vehicle (IIV+Vehicle) and AD+IIV groups (Fig. 6a, b), but no significant alterations in other CD4^+^ T cell subsets, such as Th1 (IFN-γ^+^CD4^+^), Th2 (IL-4^+^CD4^+^) and Th17 (IL-17A^+^CD4^+^) cells (Fig. S3A-C), or CD8^+^ T cells were observed in the spleen of AD+IIV mice (Fig. S4). In addition, our flow cytometry data showed that ATRA treatment decreased the frequency of mo-MΦ in the BM improved by IIV in APP/PS1 mice (Fig. S5A-B). Additionally, our findings indicate that ATRA boosted the proportion of CD4^+^Foxp3^+^ Tregs in both the APP/PS1 mice and WT mice compared with the corresponding controls (Fig. S6). Expectedly, we found that the IIV+ATRA mice performed significantly worse than the mice in the vehicle group following the Treg induction (Fig. 6c). Moreover, the IIV+ATRA mice exhibited significantly fewer crossing times, a shorter distance traveled and less time spent in the target quadrant than the vehicle mice during the spatial probe phase (Fig. 6d–f).

Interestingly, the ATRA treatment did not exacerbate cognitive function in the AD mice (Fig. S7A-D), while WT mice were slightly impaired compared with the controls (Fig. S7A-D). In addition, we observed no obvious differences in locomotor activity among these groups (Fig. S8A, B). In conclusion, these results confirm that enhanced immune suppression caused by ATRA prevents the IIV-induced protection against cognitive decline in APP/PS1 mice.

**Fig. 7:**
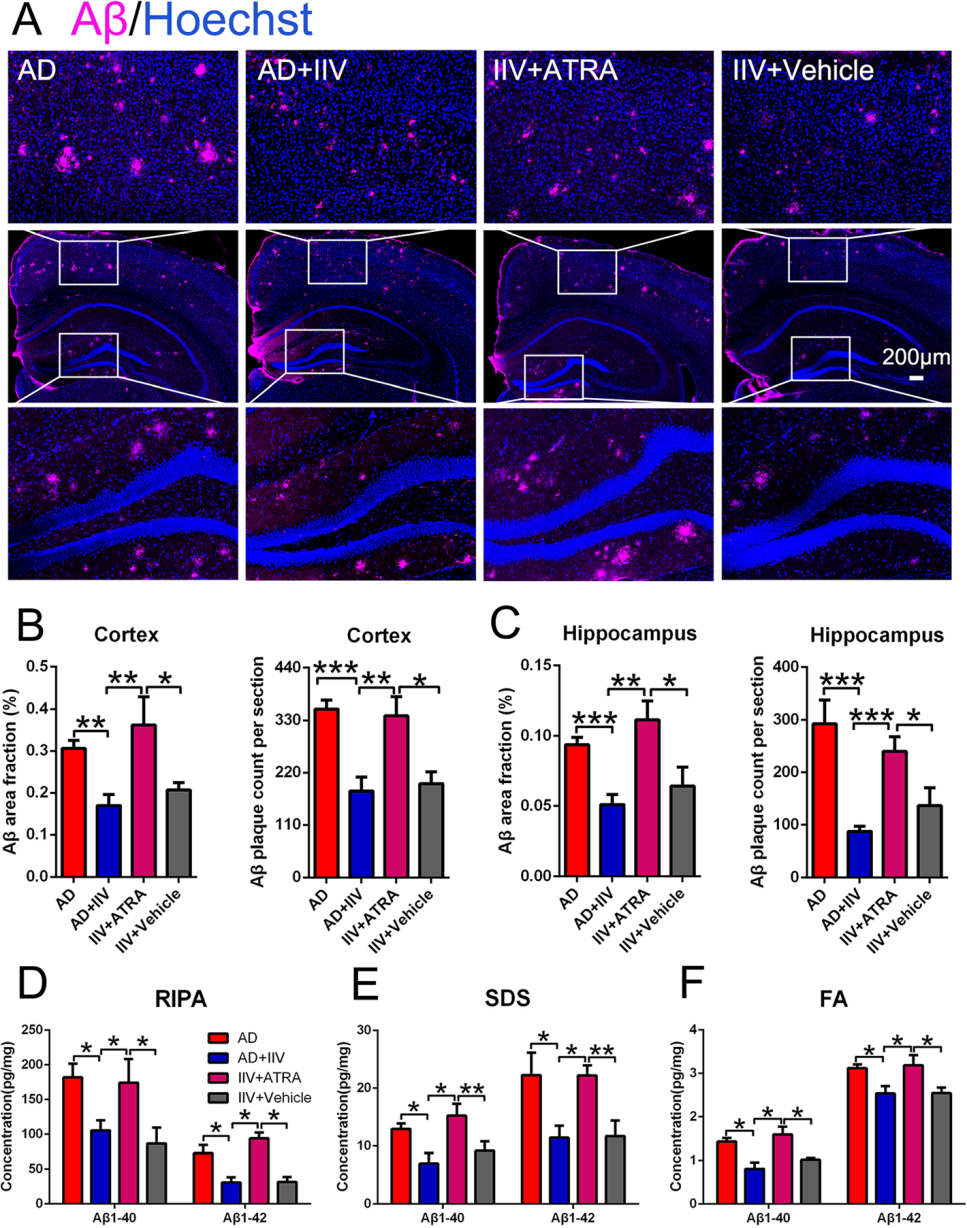
Enhanced immune suppression reversed IIV-induced improvement in Aβ pathology. **a** Representative microscopic images of the cortex and hippocampus of the AD, AD+IIV, IIV+ATRA and IIV+Vehicle mice stained for Aβ (purple) and Hoechst nuclear staining (blue) (scale bar, 200 μm). **b**, **c** Quantification of the percentage of the surface area and numbers of Aβ plaques was performed in five equidistant slices separated by 240 μm per animal (*n* = 9, mean ± SEM, one-way ANOVA and LSD post hoc analysis, *P < 0.05, **P < 0.01, ***P < 0.001). **d**–**f** ELISA was utilized to analyze the levels of soluble and insoluble Aβ_1-40_ and Aβ_1-42_ extracted with RIPA (**i**), SDS (**j**), and FA (**k**) from the cerebral cortex of 8-month-old APP/PS1 mice (*n* = 5, mean ± SEM, One-way ANOVA and LSD post hoc analysis, *P < 0.05, **P < 0.01)

**Fig. 8:**
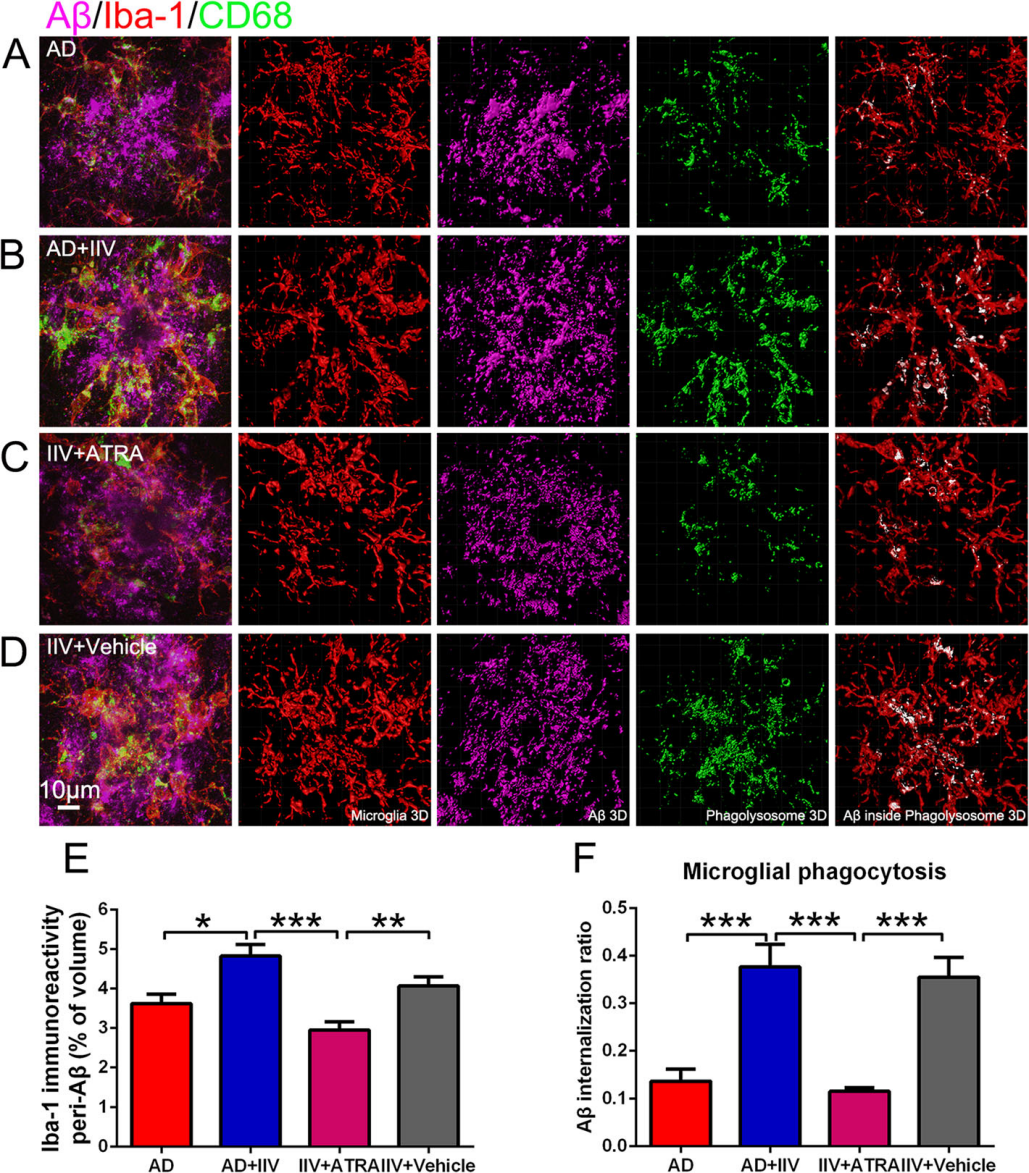
Augmenting immunosuppression impaired microglial phagocytosis improved by IIV in APP/PS1 mice. **a–d** Representative microscopy images of amyloid plaques engulfed by microglia in the brains of the AD (**a**) (*n* = 8), AD+IIV (**b**) (*n* = 8), IIV+ATRA (**c**) (*n* = 6) and IIV+Vehicle (**d**) (*n* = 6) mice. Sections were stained for Aβ (Aβ, purple), Iba-1 (microglia, red) and CD68 (phagolysosomes, green). Three-dimensional reconstruction of confocal image stacks showing red microglia, purple Aβ and green microglial phagolysosomes (colocalization of CD68 and Iba1) and merged images of microglia and white amyloid plaques within microglial phagolysosomes (colocalization of Aβ plaques and microglial phagolysosomes) (scale bar, 10 μm). **e** Quantification of the immunoactivity of Iba-1 surrounding Aβ plaques within the field in the AD, AD+IIV, IIV+ATRA and IIV+Vehicle groups (*n* > 6, mean ± SEM, one-way ANOVA and LSD post hoc analysis, *P < 0.05, **P < 0.01, ***P < 0.001). **f** Assessment of the proportions of Aβ inside microglial phagolysosomes, normalized to microglial phagolysosomes, i.e., the Aβ internalization ratio, revealed a threefold decrease in Aβ internalization in the microglia in the IIV+ATRA group compared to that in the AD+IIV group (*n* > 5, mean ± SEM, one-way ANOVA and LSD post hoc analysis, *P < 0.05)

To further corroborate the impact of Treg-induced immunosuppression on cerebral Aβ plaque burden in APP/PS1 mice, we assessed the Aβ plaque burden and microglial phagocytosis in the brain in the AD+IIV group with and without the ATRA treatment. We found that ATRA treatment prevented the decrease in Aβ plaque deposition in both the hippocampus and cortex in the AD+IIV mice (Fig. 7a–f). Consistently, the increases in microglia activation (Fig. 8e) and frequency of Aβ plaques engulfed by microglial phagolysosomes (Fig. 8f) in the AD+IIV mice were blocked by ATRA-induced immune suppression. Simultaneously, ATRA reversed the decrease in cerebral astrogliosis in IIV+ATRA mice compared with AD+IIV mice (Fig. S9).

Interestingly, different results were found in the Aβ area fraction, Aβ plaque count and total Aβ plaque area between the hippocampus and cortex in the AD mice after ATRA treatment (Fig. S10 A-D), suggesting that the effect of ATRA on Aβ deposition might be brain area specific (Fig. S10 D). Brain area sensitivity in response to ATRA treatment might be an underlying mechanism. Moreover, a ceiling effect in peripheral immunosuppression may exist in AD mice, relative to AD+IIV mice, whose immune blockade has been broken. Altogether, our data suggest that systemic immunosuppression mediated by Tregs is negatively correlated with AD pathology, implying that IIV reinforces system immune activation and enhances microglial Aβ clearance, thereby improving cognitive impairment by relieving Treg-mediated peripheral immunosuppression.

### IIV modulates the immune microenvironment to rebalance and form new homeostasis in AD brains

**Fig. 9:**
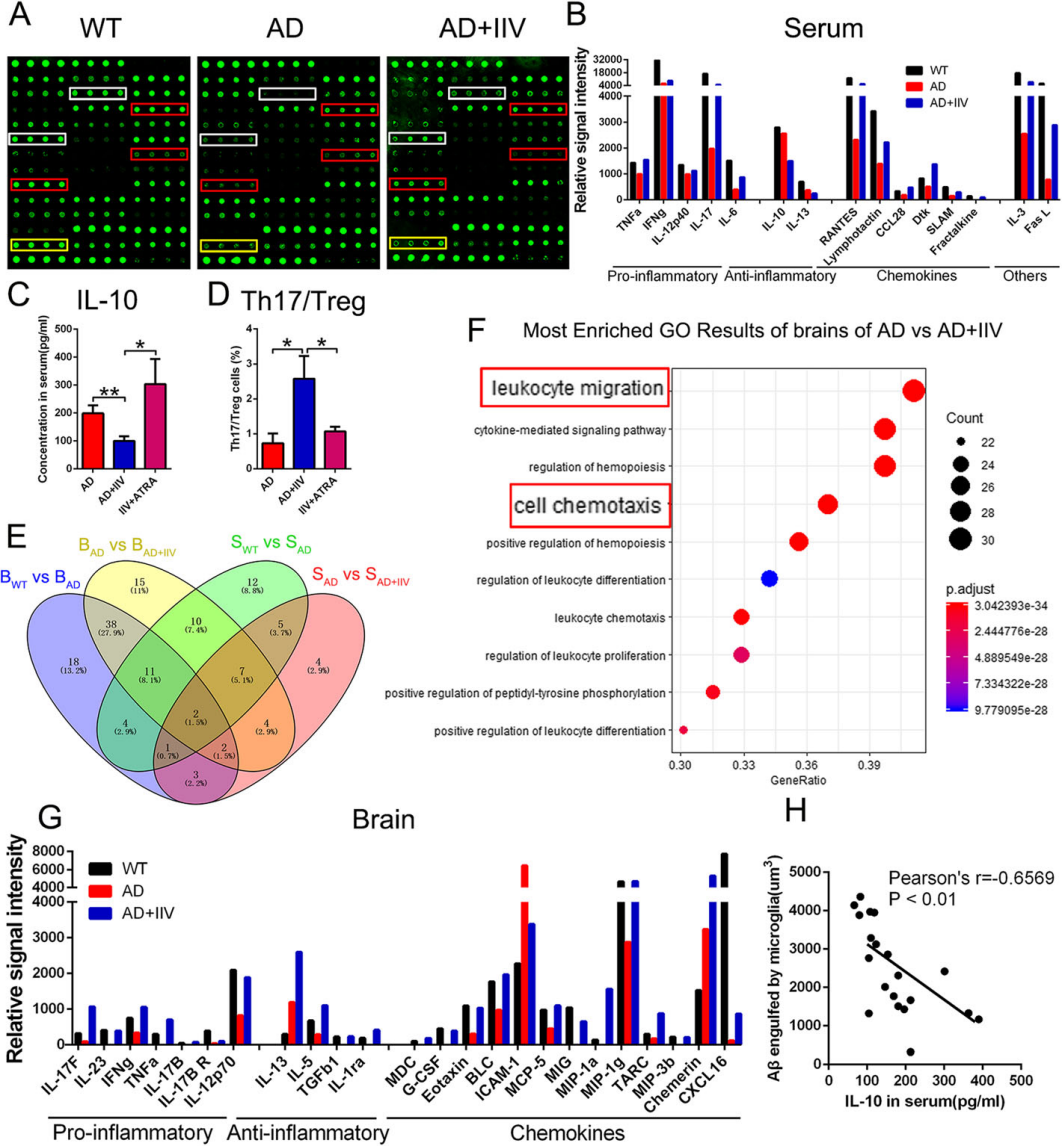
IIV modulated the immune milieu and generated new homeostasis in AD brains. **a**, **b** Cytokine array analysis of cytokine levels in the serum of WT, AD and AD+IIV mice. The red frames represent the indicated pro- and anti-inflammatory cytokines, the yellow frames represent chemokines and the white frames represent other cytokines (**a**) (*n* = 1, mix of 4 mice/group). Trend graph of the relative signal intensity presented in different categories (**b**) (*n* = 1, mix of 4 mice/group) as proinflammatory, anti-inflammatory, chemokines and other cytokines. **c**, **d** IL-10 levels in serum (**c**) and the ratio of Th17 to Tregs in the spleen (**d**) in AD, AD+IIV and IIV+ATRA mice (*n* ≥ 6, mean ± SEM, One-way ANOVA, *P < 0.05). **e** Venn diagram showing the overlap among the indicated cytokines in the serum and brain tissue in the WT, AD and AD+IIV groups. **f** GO functional enrichment analysis of the biological process of the indicated cytokines obtained from the brains of AD mice and AD+IIV mice. Representative enriched pathways mainly concentrated in leukocyte migration and cell chemotaxis. **g** Representative cytokine trend graph of the relative signal intensity in the brains of AD and AD+IIV mice presented as proinflammatory, anti-inflammatory and chemokines (*n* = 1, mix of 4 mice/group). **h** Correlation analysis between the level of serum IL-10 and the volume of Aβ engulfed by microglia was performed, and a negative relationship was found (Pearson’s *r* = − 0.6569, *P* < 0.01)

Emerging evidence suggests that the immune microenvironment of AD brains is dysfunctional [36, 37]. In the present study, IIV broke Treg-mediated systemic immune suppression, followed by the activation of brain-resident immune cells. However, the connections between the attenuation of peripheral immune tolerance and microglia activation remain unknown. To further investigate the link between the peripheral and brain immune system and their cross talk, the cytokine profiles in the serum and brain lysates of WT mice, AD mice and AD+IIV mice were analyzed using a RayBio Mouse Cytokine Antibody Array. The findings suggest that most serum proinflammatory cytokines, chemokines and partial cytokines in the AD mice were lower than those in the WT group and that the IIV treatment boosted the expression of both proinflammatory cytokines and chemokines in AD mice, reaching the levels observed in WT mice (Fig. 9a, b). Notably, IIV might downregulate the anti-inflammatory cytokine levels in the serum of AD mice (Fig. 9a, b), for example, IL-10, which is a Treg-secreted cytokine [38, 39], showed a proinflammatory state in the periphery. The ELISA test confirmed that IIV decreased while ATRA elevated IL-10 expression (Fig. 9c). Further analysis revealed that the Th17/Treg ratio was obviously higher in AD+IIV mice and that this increase could be reversed by the ATRA-induced peripheral immune tolerance (Fig. 9d). Collectively, these observations suggest that IIV immunization might alter the peripheral immunosuppression milieu, reduce anti-inflammatory cytokine levels and increase proinflammatory cytokine levels, thereby generating new homeostasis in AD brains.

The characterization of the differences in serum and brain protein expression, using an unbiased cluster analysis, revealed 15 distinct serum expression profiles and 53 distinct brain expression profiles among the WT, AD and AD+IIV groups (Fig. 9e), suggesting a greater effect on the brain than the periphery. Moreover, the GO enrichment analysis of distinct brain proteins indicated that the functions of these differentially expressed proteins were mainly related to leukocyte migration and cell chemotaxis (Fig. 9f). Furthermore, the RayBio Mouse Cytokine Antibody Array suggested that most pro- and anti-inflammatory cytokines and chemokines showed an increasing trend in the brain of AD+IIV mice compared with those in the brain of AD mice (Fig. 9g). Notably, the slight inverse correlation between serum IL-10 and Aβ endocytosis suggests that the transient decline in Treg activity may play a role in the IIV-induced cognitive improvement in AD mice (Fig. 9h).

Altogether, our data suggest that a distinct immune protein profile exists in both the periphery and brain that is suggestive of leukocyte migration, chemotaxis, and differentiation in IIV-treated APP/PS1 mice.

## Discussion

In this study, we showed that a nonspecific immune challenge with IIV-modified AD pathology and improved cognitive performance in the early phase of AD in a mouse model. Specifically, IIV broke Treg-mediated peripheral immunosuppression and reduced the levels of anti-inflammatory factors by boosting the peripheral immune system. However, the augmentation of systemic Treg activity by a pharmacological approach abolishes the protective effects on disease pathology and memory functions. Most interestingly, boosting the systemic immune response might increase both pro- and anti-inflammatory factor release in the brain and built a novel immunological milieu in which microglia activation and Aβ plaque clearance occur. The beneficial effects of IIV on AD pathology suggest that IIV can be developed as a new immunotherapy strategy for the treatment of AD at the early stages.

Emerging evidence has shown that immune system dysfunction may contribute to AD progression [9, 31, 32]. For example, alteration in the intestinal flora of AD patients has been reported to contribute to AD [40]. Thus, researchers have suggested that immune intervention may be a better direction for prevention and therapy [32]. Importantly, patients with heart failure vaccinated with influenza vaccine three times had a significant lower risk of dementia than those who were not vaccinated [41, 42]. Therefore, we hypothesized that multiple IIV might be effective in an AD mouse model. Unexpectedly, IIV significantly improved the Aβ plaque burden and cognitive deficits in APP/PS1 mice following the revitalization of the systemic immune response and innate immunity in the brain. Studies involving anti-inflammatory and immunosuppressive therapies have failed in clinical trials [43–45], which inspired us to consider that breaking the peripheral immune tolerance, especially at early disease stages, might be a novel therapeutic intervention for AD.

Tregs are CD4 positive T-lymphocyte subsets that can maintain peripheral immune tolerance [25, 46, 47]. Previous and our studies have reported elevated Treg counts in AD patients and an Aβ-driven AD mouse model [17, 27, 48], supporting the notion that aging and AD are characterized by immune tolerance and/or immune senescence [13, 49]. In the present study, the systemic reduction in peripheral Tregs after the IIV treatment was associated with the mitigation of the disease pathology and cognitive impairments. Importantly, the beneficial effects induced by IIV are partially prevented by ATRA-induced Treg amplification. Combined with the independent studies conducted in Michal Schwartz’s laboratory [15, 50], these data suggest that the beneficial effects induced by IIV are mediated, at least partially, via interference with systemic Treg activity.

Recently, conflicting results regarding the role of systemic Tregs in AD pathology have been reported. Consistent with the conclusion reported by Michal Schwartz’s group, breaking Treg-driven immunosuppression in the periphery is associated with improvement in Alzheimer-like amyloid pathology. These beneficial effects were confirmed by the transient deletion of Tregs [13] or ATRA-induced Treg amplification in the present study. However, several studies yielding data contradictory to our data have shown that expanding and activating Tregs in the periphery through chronic IL-2 treatment have beneficial effects on cognition during both early AD stages and established pathological stages [33, 51]. Moreover, systemic Treg transplantation into an AD mouse model improved cognitive functions and reduced Aβ deposition [52] However, in these three studies related to Treg amplification, different findings were observed in terms of Aβ clearance and plaque-related microglia in the hippocampus. Notably, increases in Tregs in the brain were found both after Treg amplification (IL-2 treatment) and transient systemic Treg depletion in two independent studies [13, 51]. Therefore, whether a common mechanism underlying the recruitment of Treg cells into the brain exists following the amplification or depletion of systemic Tregs remains a key question that needs to be further explored.

Notably, the analysis of the potential immunological mechanisms using a protein microarray revealed distinct immune protein expression profiles between the serum and brain tissue but identified consistent leukocyte migration and chemotaxis regulation in these two areas as the top GO enrichment categories. Together with our previous report [24] showing that IIV recruits T lymphocytes to the choroid plexus and promotes hippocampal neurogenesis, these results suggest that T lymphocytes might be recruited to the brain via the CP gateway to exert the functional modulation of microglial activation and phagocytosis. Moreover, consistent with a previous report [53], an inverse correlation was observed between the systemic CD4^+^CD25^+^Foxp3^+^ Treg levels and the count of CD11b^+^CD45^high^ monocytes in the BM in AD+IIV mice. Therefore, whether increased monocytes result in systemic Treg reduction still needs to be determined to assess the mechanisms underlying such cross talk.

The distinct protein profiles suggest that the peripheral and central cytokine networks play distinct roles in the immunotherapy of AD. In particular, we first reported that brain pro- and anti-inflammatory cytokines showed an increasing trend after 5 IIV treatments, indicating that a mixture of immune training and immune tolerance is induced by preliminary IIV stimulation. Although the immune protein expression profiles in the brain induced by the 5 IIV treatment in this study differ from those in a recent report of immune tolerance following 5 LPS injections [54], a mixture of immune training and tolerance and immune tolerance alone exerted the same beneficial effects in reducing AD pathology [54]. Further analysis indicated that the cytokines with different changes between the brain and serum are not completely consistent. Similarly, Zarif et al. [55] demonstrated that T lymphocytes purified from the spleen and choroid plexus showed different mRNA transcriptomic profiles as reflected by single-cell RNA seq analyses. The above studies suggest distinguished immune cytokine profiles between the periphery and the brain. In addition, many conflicting findings strongly confirm the complex role of the cytokine and chemokine profiles associated with neurodegenerative diseases, especially AD.

Recently, several studies have suggested that the frequencies of Th17 and the Th17/Treg ratio are enhanced in AD and/or MCI patients [56, 57], which seems to contradict our results. However, our data show that the proportion of CD4^+^IL-17A^+^ Th17 cells was not altered among the WT, WT+IIV, AD and AD+IIV groups. CD4+Foxp3+ Tregs were significantly increased in AD group, relative to WT group, whereas these increases were prevented in AD group after the IIV treatment. Therefore, our results suggest that the higher IIV-induced Th17/Treg ratio is due to the decreased proportion of Tregs rather than higher Th17 proportion. Regarding the different Th17 proportion in AD patients reported in recent studies [56, 57] and our results, a possible explanation for the differences is related to the different research subjects and immune cells (PBMC vs. spleen cells). It could be valuable to obtain similar data (Th17/Treg ratio) using different transgenic AD mouse models (3xFAD etc.) in the future.

Microglia, which are the resident immune cells in the brain, have been suggested to become anergic and inefficient in phagocytosing Aβ deposits during AD progression [58]. In fact, although IIV is a nonspecific immune modulator, it enhanced Aβ phagocytosis by microglia as reflected by the increased expression of the scavenger receptor CD68. Furthermore, the increase in cerebral chemokines may drive more microglia to be recruited to surround and engulf Aβ plaques. These results suggest that IIV boosted systemic immune responses by revitalizing the phagolysosome activity of microglia and promoting Aβ clearance. The epigenetics associated with immune memory and the direct regulatory mechanism targeting microglia remain to be determined in the future.

In conclusion, we found that IIV immunization could antagonize the immunosuppression of Tregs by reducing the Tregs levels and increasing mo-MΦ to break peripheral immunosuppression, resulting in alterations in the peripheral-derived signals associated with disease progression. The accumulation of these disease-related peripheral signals in the brain may affect microglia activation, thereby rebalancing the disordered immune milieu in the brain. Collectively, our findings suggest that in addition to preventing influenza, multiple influenza vaccinations during the early stage of AD, may be regarded as a new, inexpensive and easily available effective therapeutic intervention for AD, especially among elderly people, who are recommended to receive IIV vaccination in China and many other countries [59].

## Conclusions

In summary, IIV vaccination during the early stage of AD is sufficient to rescue amyloidosis and ameliorate cognitive deficits in APP/PS1 mice. Mechanistically, this progress may be associated with broken Treg-mediated peripheral immune suppression, a boosted peripheral immune system and a rebalanced immunological milieu in the brain in which microglia activation and Aβ plaque clearance occur. Our findings strongly suggest that nonspecific immunomodulator IIV vaccination in the early stages can be developed as a new immunotherapy strategy for AD.

## Supplementary information

**Additional file 1: Figure S1.** No significant effect of IIV on spontaneous locomotor activity. The distance travelled overall, in the periphery and center arena (A), entries (B) and staying durations (C) in the center arena of the WT (*n* = 8), WT+IIV (*n* = 14), AD (*n* = 15) and AD+IIV (*n* = 16) mice during the open field test were analyzed (mean ± SEM, one-way ANOVA and LSD post hoc analysis).

**Additional file 2: Figure S2.** IIV treatment reversed peripheral mo-MΦ in BM in APP/PS1 mice. (A and B) Representative flow cytometric plots (A) and quantitative analysis (B) of the frequencies of CD45^+^CD11b^+^ monocyte-derived macrophages of BM cells analyzed by flow cytometry (*n* > 10, mean ± SEM, one-way ANOVA and LSD post hoc test, *P < 0.05, ***P < 0.001).

**Additional file 3: Figure S3.** ATRA treatment did not affect the proportions of other T cell subtypes in IIV-treated APP/PS1 mice. (A-C) Intracellular staining of CD4^+^ splenocytes producing IFN-γ (Th1) (A), IL-4 (Th2) (B) and IL-17A (Th17) cells (C) is presented as flow cytometry plots, and their quantitative analysis is shown on the right (*n* > 5, mean ± SEM, one-way ANOVA and LSD post hoc test).

**Additional file 4: Figure S4.** ATRA treatment did not significantly affect CD8^+^ T cells in IIV-treated APP/PS1 mice. Quantitative analysis of the frequencies of CD8^+^ T cells in the spleen of ATRA-treated AD+IIV mice by flow cytometry showed no significant effect of the ATRA treatment on the proportions of CD8^+^ T cells in the spleen of AD+IIV mice (*n* > 4, mean ± SEM, one-way ANOVA and LSD post hoc test).

**Additional file 5: Figure S5.** ATRA reversed the peripheral increase in mo-MΦ induced by IIV in BM in APP/PS1 mice. (A and B) Representative flow cytometric plots (A) and quantitative analysis (B) of the frequencies of CD45^+^CD11b^+^ monocyte-derived macrophages in BM cells analyzed by flow cytometry (*n* > 10, mean ± SEM, one-way ANOVA and LSD post hoc test, *P < 0.05, **P < 0.01).

**Additional file 6: Figure S6.** ATRA enhanced the frequency of CD4^+^Foxp3^+^ Tregs in APP/PS1 mice. (A and B) Representative flow cytometry plots (A) and quantitative analysis (B) of the proportions of CD4^+^Foxp3^+^ Treg splenocytes reflecting that the frequencies of Tregs in the AD mice were higher than those in the WT mice. Similarly, the ATRA treatment increased the ratio of Tregs (*n* > 6, mean ± SEM, one-way ANOVA and LSD post hoc test, **P < 0.01).

**Additional file 7: Figure S7.** No effect of ATRA on cognitive function in APP/PS1 animals. (A) MWM analysis of the escape latency (A) of the WT+Vehicle (*n* = 6), WT+ATRA (*n* = 6), AD+Vehicle (*n* = 6) and AD+ATRA mice (*n* = 6) (mean ± SEM, two-way repeated-measures ANOVA and LSD post hoc test, *P < 0.05, **P < 0.01, ***P < 0.001) during the acquisition phase. (B-D) Average numbers of platform crossings (C), percentage of time and distance spent (D) in the target quadrant in each group and their representative traces (B) during the spatial probe phase (*n* = 6) (mean±SEM, one-way ANOVA and LSD post hoc analysis; *P < 0.05, **P < 0.01).

**Additional file 8: Figure S8.** No evident effect on locomotor activity after enhancing peripheral immune suppression in IIV-treated APP/PS1 mice. (A and B) Open field test was performed to analyze the distance travelled overall and in the peripheral and center arenas (A) and total rearing activities (B) of the AD, AD+IIV, IIV+ATRA and IIV+Vehicle mice (*n* > 5, mean ± SEM, one-way ANOVA and LSD post hoc test).

**Additional file 9: Figure S9.** Augmented peripheral immunosuppression reversed the immunoreactivity of GFAP reduced by IIV in APP/PS1 mice. (A-D) Representative microscopy images of GFAP (red) and Hoechst nuclear staining (blue) (scale bar, 200 μm) in the AD (A), AD+IIV (B), IIV+ATRA (C) and IIV+Vehicle (D) mice. (E) Quantification of GFAP immunostaining in the hippocampus in the four groups. (*n* > 6, one-way ANOVA and LSD post hoc analysis, *P < 0.05, **P < 0.01).

**Additional file 10: Figure S10.** ATRA increased Aβ deposition in the hippocampus of APP/PS1 mice. (A and B) Representative microscopy images of the cortex and hippocampus of AD (A) and AD+ATRA mice (B) stained for Aβ plaques (purple) and Hoechst nuclear staining (blue) (scale bar, 200 μm). (C and D) Quantification of the area fraction, numbers and total area of Aβ plaques was performed in five equidistant slices separated by 240 μm per animal (*n* = 6, mean ± SEM, Student’s t-test, **P < 0.01, ***P < 0.001). (E-G) Biochemical analysis of soluble and insoluble Aβ1-40 and Aβ1-42 extracted with RIPA (E), SDS (F) and FA (G) from the cerebral cortex of 8-month-old APP/PS1 mice by ELISA (*n* = 5, mean ± SEM, one-way ANOVA and LSD post hoc analysis, *P < 0.05).

## Data Availability

All data and materials supporting the conclusions of this article are presented in the manuscript and its additional file.

## Abbreviations

AD: Alzheimer’s disease
APP: Amyloid precursor protein
ATRA: All-trans retinoic acid
Aβ: β-amyloid
BCA: Bicinchoninic acid
BFA: Brefeldin-A
DMSO: Dimethyl sulfoxide
ELISA: Enzyme-linked immunosorbent assay
GO: Gene Ontology
HBSS: Hank’s balanced salt solution
IIV: Inactivated influenza vaccine
LPS: Lipopolysaccharide
mo-MΦ: Monocyte-derived macrophages
NFTs: Neurofibrillary tangles
PBS: Phosphate buffer saline
PMA: Phorbol 12-myristate 13-acetate
PS1: Presenilin 1
Treg: Regulatory T cell
