# Supplementary figures and images for: Influenza vaccination in early Alzheimer’s disease rescues amyloidosis and ameliorates cognitive deficits in APP/PS1 mice by inhibiting regulatory T cells
Source: J Neuroinflammation. 2020 Feb 19;17:65. doi: 10.1186/s12974-020-01741-4 (PMC7029575; doi:10.1186/s12974-020-01741-4)

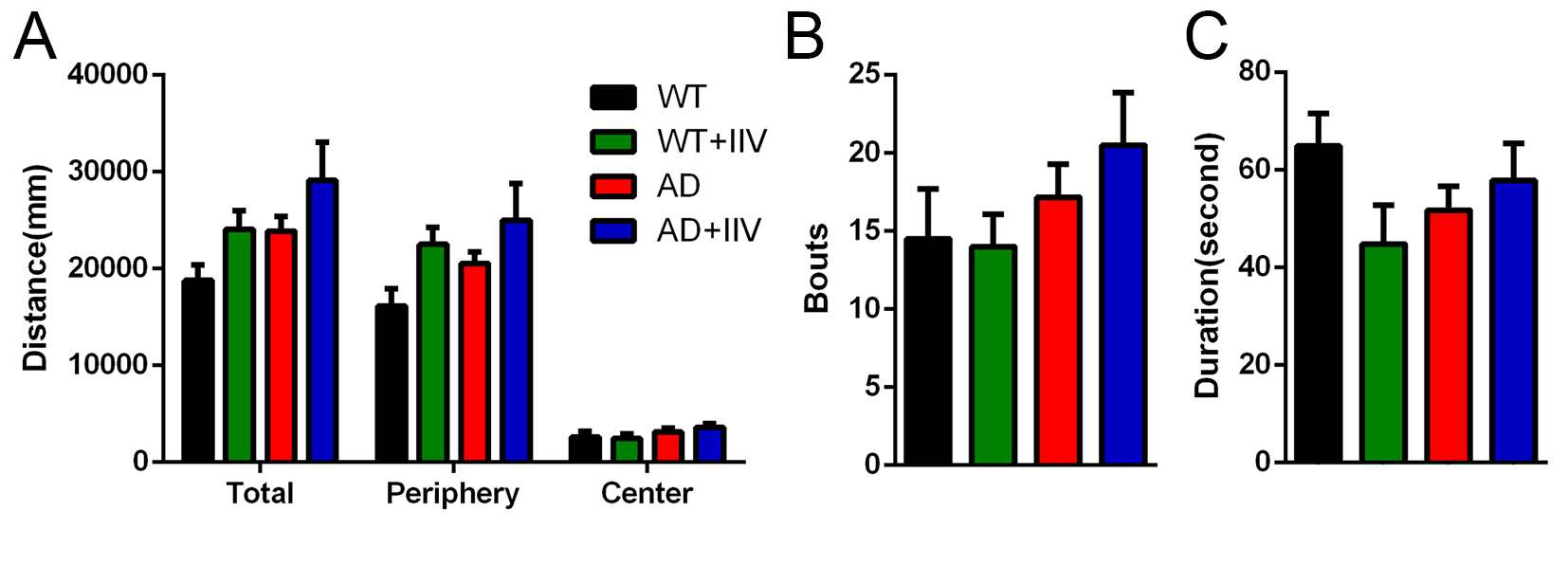

Supplement: Supplementary file 1 — Additional file 1: Figure S1. No significant effect of IIV on spontaneous locomotor activity. The distance travelled overall, in the periphery and center arena (A), entries (B) and staying durations (C) in the center arena of the WT (n = 8), WT+IIV (n = 14), AD (n = 15) and AD+IIV (n = 16) mice during the open field test were analyzed (mean ± SEM, one-way ANOVA and LSD post hoc analysis). [file 12974_2020_1741_MOESM1_ESM.tif]

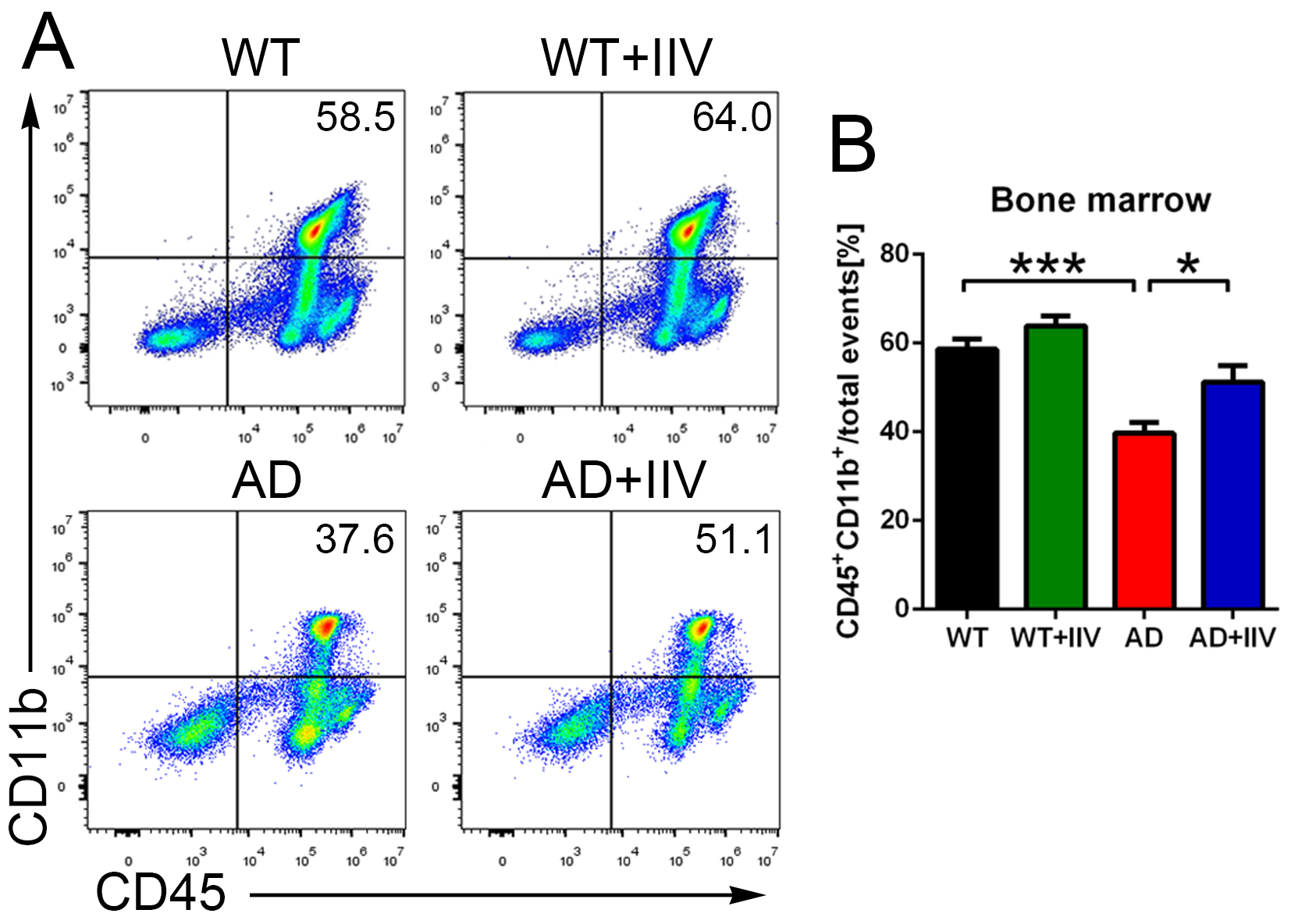

Supplement: Supplementary file 2 — Additional file 2: Figure S2. IIV treatment reversed peripheral mo-MΦ in BM in APP/PS1 mice. (A and B) Representative flow cytometric plots (A) and quantitative analysis (B) of the frequencies of CD45+CD11b+ monocyte-derived macrophages of BM cells analyzed by flow cytometry (n > 10, mean ± SEM, one-way ANOVA and LSD post hoc test, *P < 0.05, ***P < 0.001). [file 12974_2020_1741_MOESM2_ESM.tif]

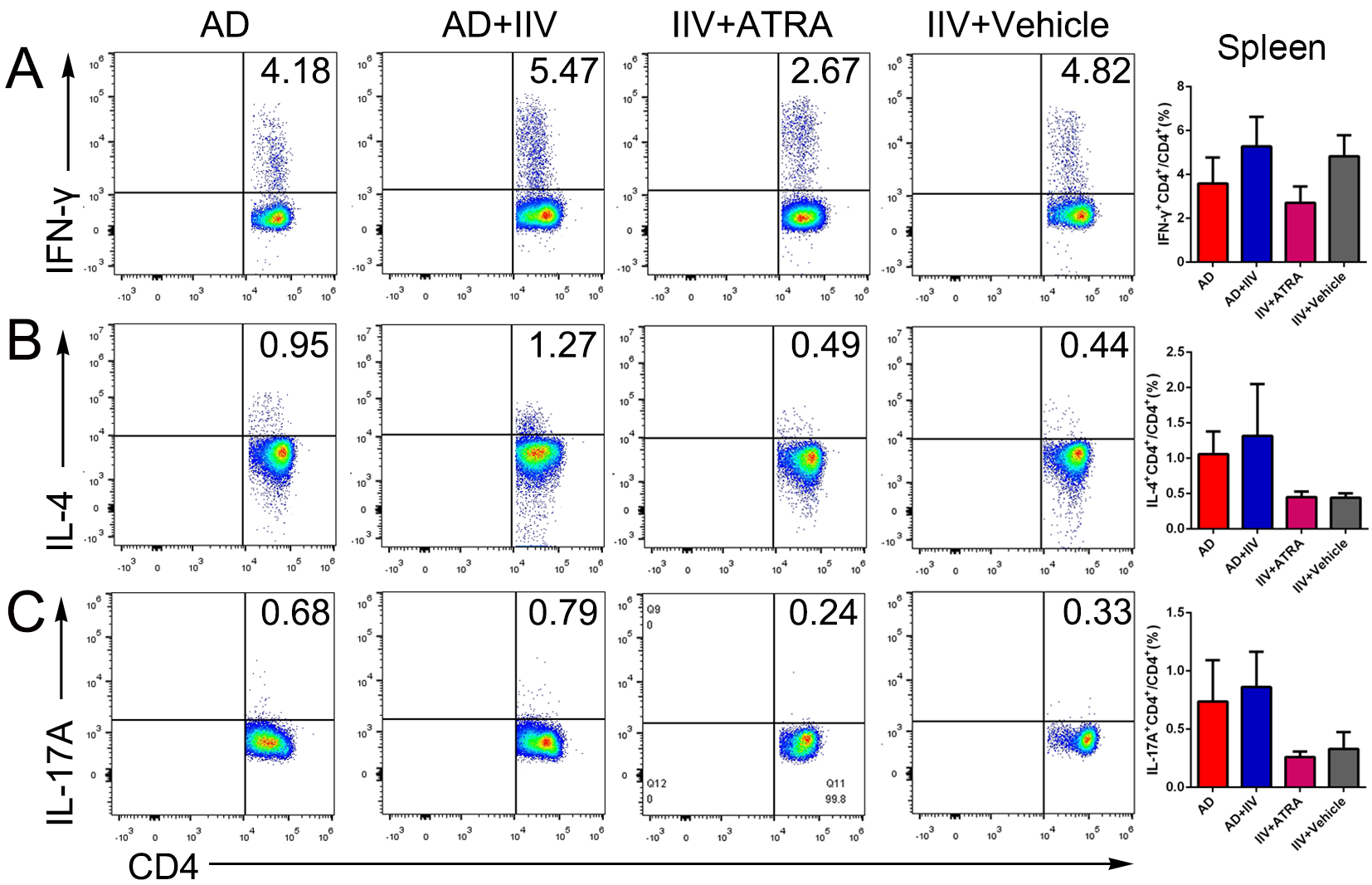

Supplement: Supplementary file 3 — Additional file 3: Figure S3. ATRA treatment did not affect the proportions of other T cell subtypes in IIV-treated APP/PS1 mice. (A-C) Intracellular staining of CD4+ splenocytes producing IFN-γ (Th1) (A), IL-4 (Th2) (B) and IL-17A (Th17) cells (C) is presented as flow cytometry plots, and their quantitative analysis is shown on the right (n > 5, mean ± SEM, one-way ANOVA and LSD post hoc test). [file 12974_2020_1741_MOESM3_ESM.tif]

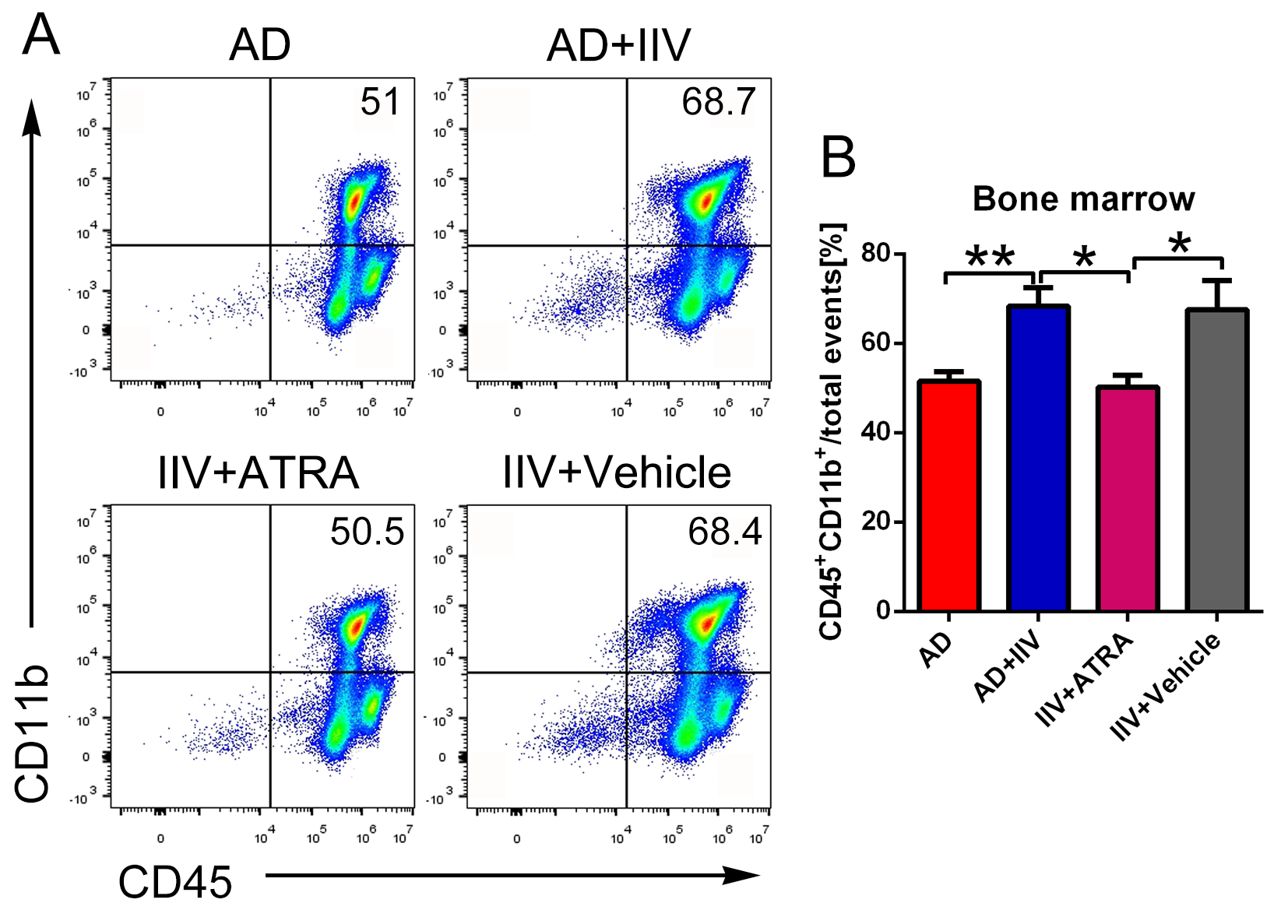

Supplement: Supplementary file 5 — Additional file 5: Figure S5. ATRA reversed the peripheral increase in mo-MΦ induced by IIV in BM in APP/PS1 mice. (A and B) Representative flow cytometric plots (A) and quantitative analysis (B) of the frequencies of CD45+CD11b+ monocyte-derived macrophages in BM cells analyzed by flow cytometry (n > 10, mean ± SEM, one-way ANOVA and LSD post hoc test, *P < 0.05, **P < 0.01). [file 12974_2020_1741_MOESM5_ESM.tif]

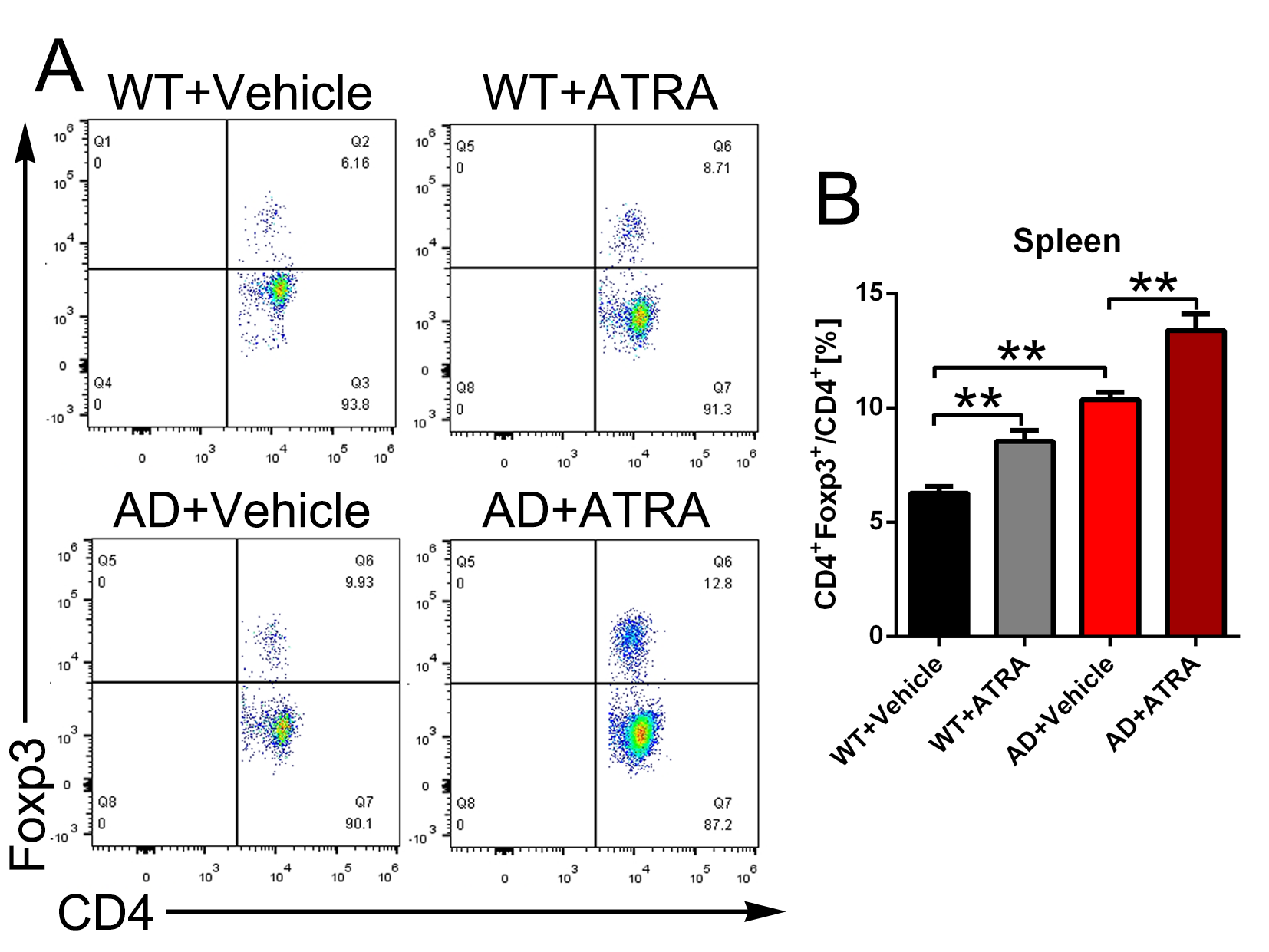

Supplement: Supplementary file 6 — Additional file 6: Figure S6. ATRA enhanced the frequency of CD4+Foxp3+ Tregs in APP/PS1 mice. (A and B) Representative flow cytometry plots (A) and quantitative analysis (B) of the proportions of CD4+Foxp3+ Treg splenocytes reflecting that the frequencies of Tregs in the AD mice were higher than those in the WT mice. Similarly, the ATRA treatment increased the ratio of Tregs (n > 6, mean ± SEM, one-way ANOVA and LSD post hoc test, **P < 0.01). [file 12974_2020_1741_MOESM6_ESM.tif]

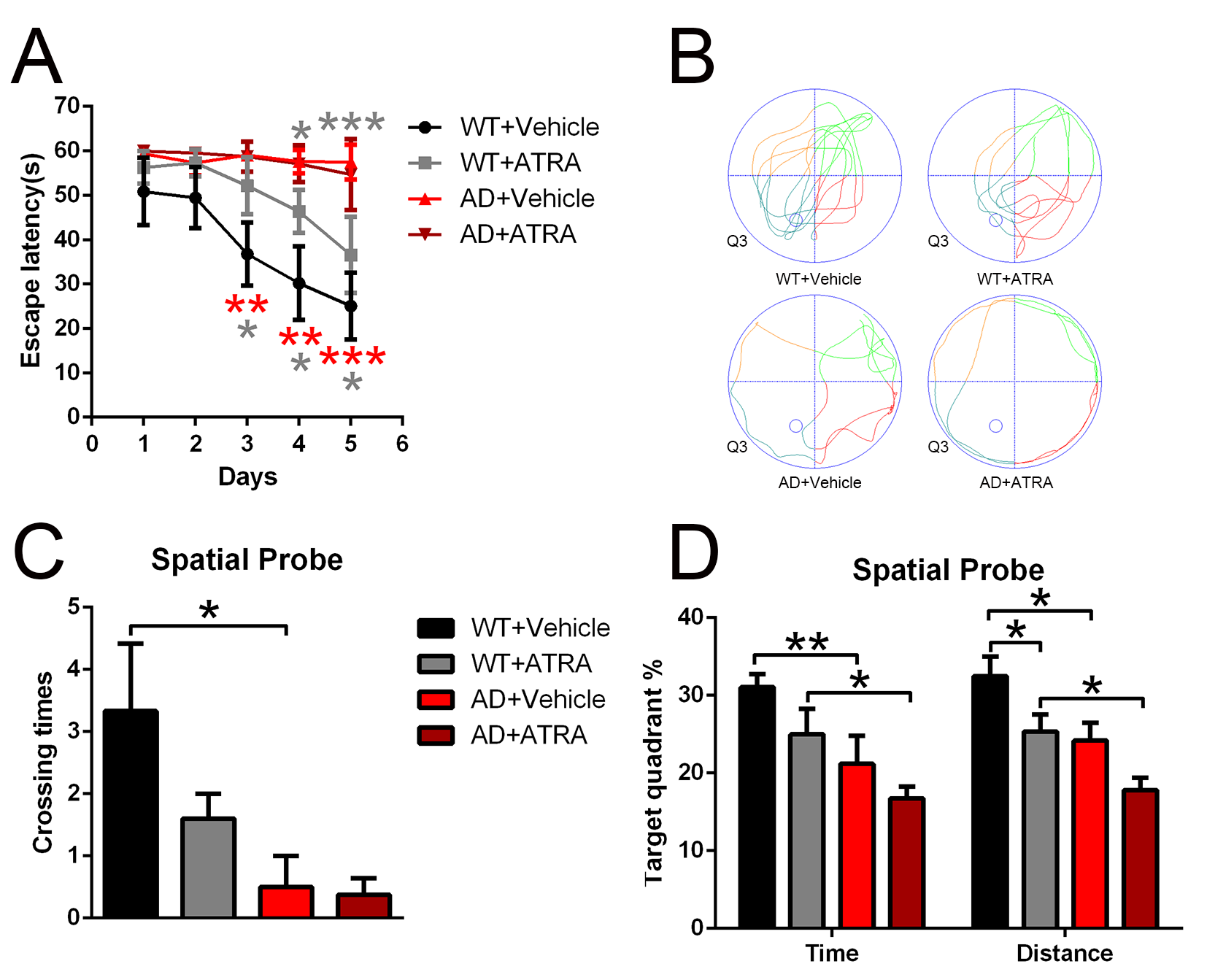

Supplement: Supplementary file 7 — Additional file 7: Figure S7. No effect of ATRA on cognitive function in APP/PS1 animals. (A) MWM analysis of the escape latency (A) of the WT+Vehicle (n = 6), WT+ATRA (n = 6), AD+Vehicle (n = 6) and AD+ATRA mice (n = 6) (mean ± SEM, two-way repeated-measures ANOVA and LSD post hoc test, *P < 0.05, **P < 0.01, ***P < 0.001) during the acquisition phase. (B-D) Average numbers of platform crossings (C), percentage of time and distance spent (D) in the target quadrant in each group and their representative traces (B) during the spatial probe phase (n = 6) (mean±SEM, one-way ANOVA and LSD post hoc analysis; *P < 0.05, **P < 0.01). [file 12974_2020_1741_MOESM7_ESM.tif]

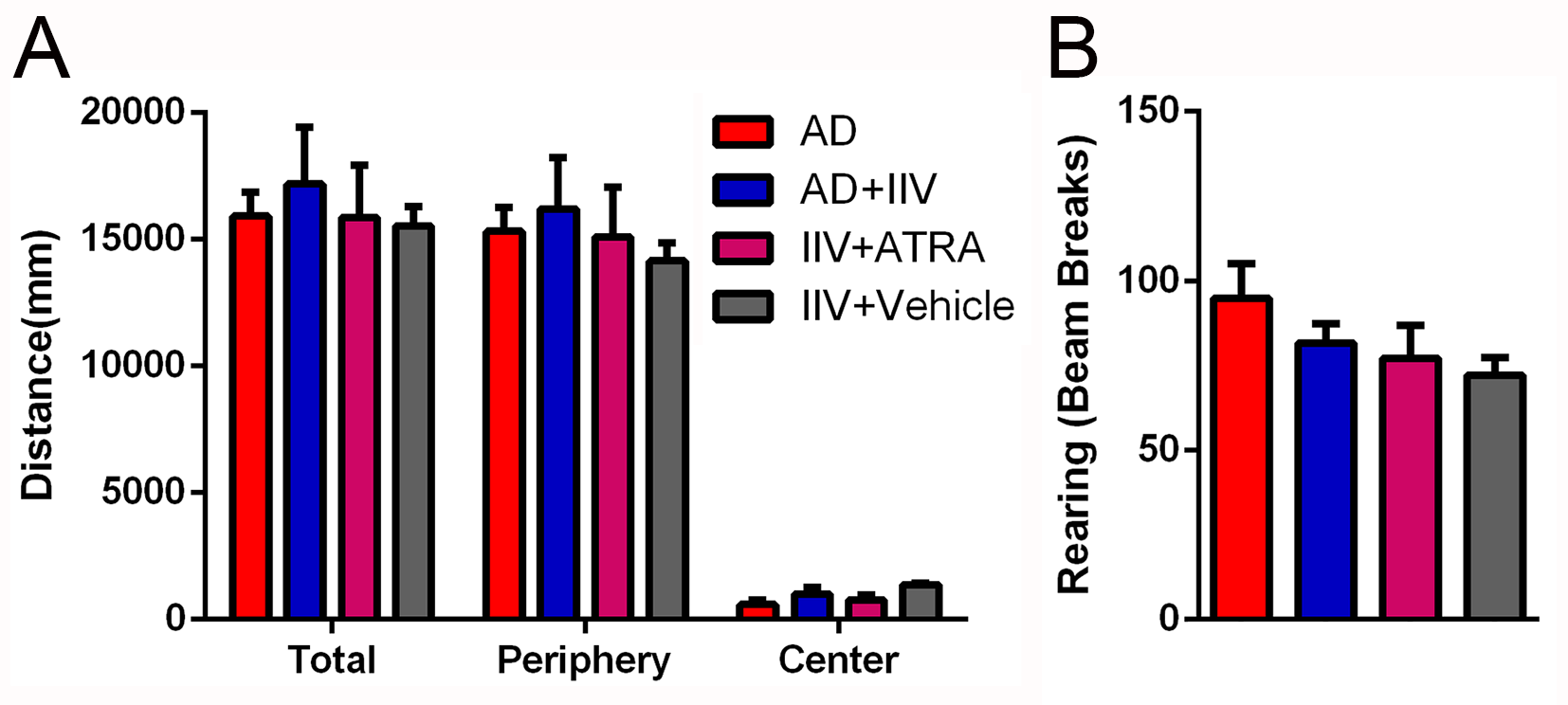

Supplement: Supplementary file 8 — Additional file 8: Figure S8. No evident effect on locomotor activity after enhancing peripheral immune suppression in IIV-treated APP/PS1 mice. (A and B) Open field test was performed to analyze the distance travelled overall and in the peripheral and center arenas (A) and total rearing activities (B) of the AD, AD+IIV, IIV+ATRA and IIV+Vehicle mice (n > 5, mean ± SEM, one-way ANOVA and LSD post hoc test). [file 12974_2020_1741_MOESM8_ESM.tif]

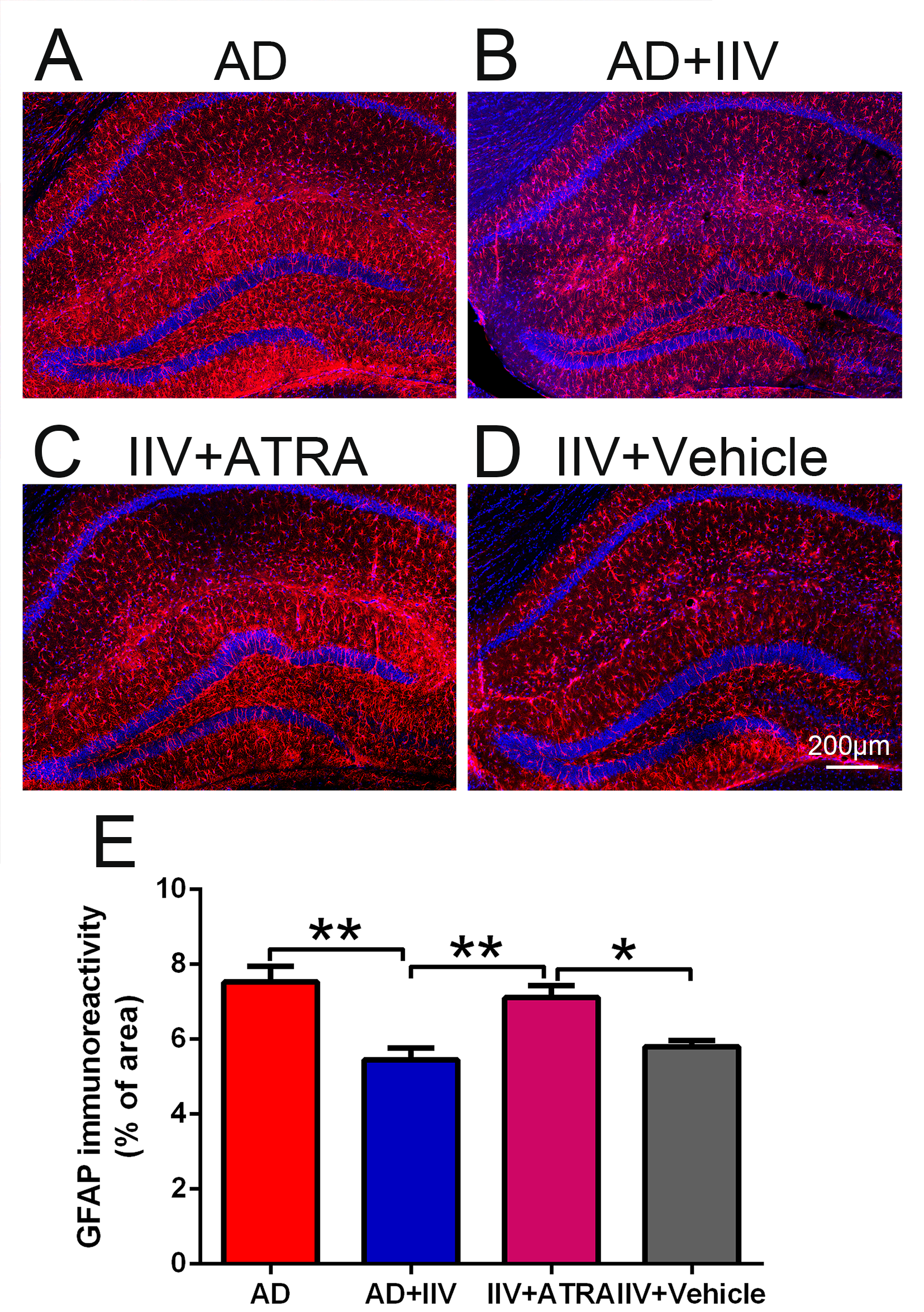

Supplement: Supplementary file 9 — Additional file 9: Figure S9. Augmented peripheral immunosuppression reversed the immunoreactivity of GFAP reduced by IIV in APP/PS1 mice. (A-D) Representative microscopy images of GFAP (red) and Hoechst nuclear staining (blue) (scale bar, 200 μm) in the AD (A), AD+IIV (B), IIV+ATRA (C) and IIV+Vehicle (D) mice. (E) Quantification of GFAP immunostaining in the hippocampus in the four groups. (n > 6, one-way ANOVA and LSD post hoc analysis, *P < 0.05, **P < 0.01). [file 12974_2020_1741_MOESM9_ESM.tif]

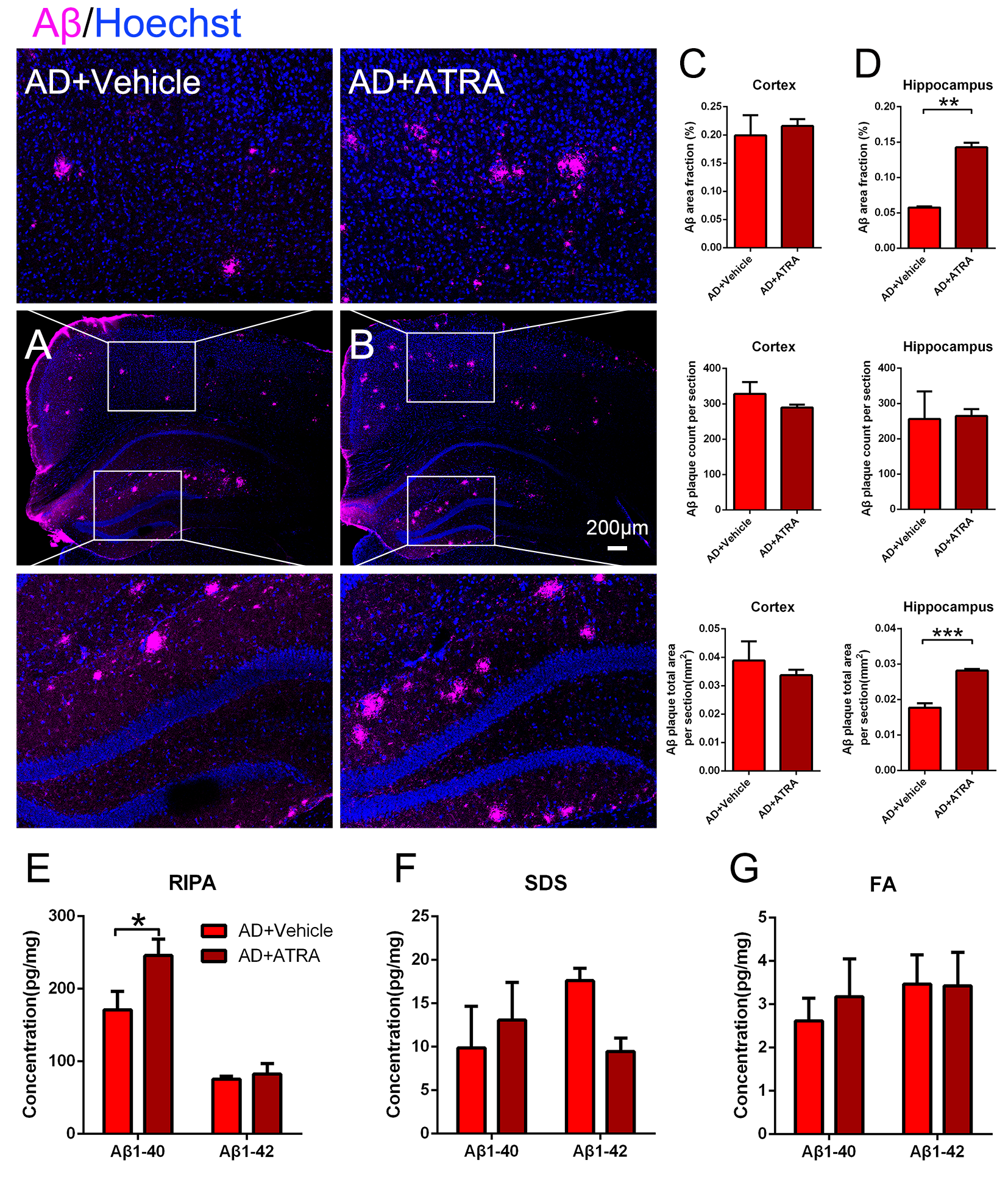

Supplement: Supplementary file 10 — Additional file 10: Figure S10. ATRA increased Aβ deposition in the hippocampus of APP/PS1 mice. (A and B) Representative microscopy images of the cortex and hippocampus of AD (A) and AD+ATRA mice (B) stained for Aβ plaques (purple) and Hoechst nuclear staining (blue) (scale bar, 200 μm). (C and D) Quantification of the area fraction, numbers and total area of Aβ plaques was performed in five equidistant slices separated by 240 μm per animal (n = 6, mean ± SEM, Student’s t-test, **P < 0.01, ***P < 0.001). (E-G) Biochemical analysis of soluble and insoluble Aβ1-40 and Aβ1-42 extracted with RIPA (E), SDS (F) and FA (G) from the cerebral cortex of 8-month-old APP/PS1 mice by ELISA (n = 5, mean ± SEM, one-way ANOVA and LSD post hoc analysis, *P < 0.05). [file 12974_2020_1741_MOESM10_ESM.tif]
